# Intuitive and Efficient Approach to Determine the Band Structure of Covalent Organic Frameworks from Their Chemical Constituents

**DOI:** 10.1021/acs.jctc.3c01302

**Published:** 2024-02-02

**Authors:** Changchun Ding, Xiaoyu Xie, Linjiang Chen, Alessandro Troisi

**Affiliations:** † School of Science, Xihua University, Chengdu 610039, China; ‡ Department of Chemistry, University of Liverpool, Liverpool L69 3BX, U.K.; § School of Chemistry and School of Computer Science, University of Birmingham, Birmingham B15 2TT, U.K.

## Abstract

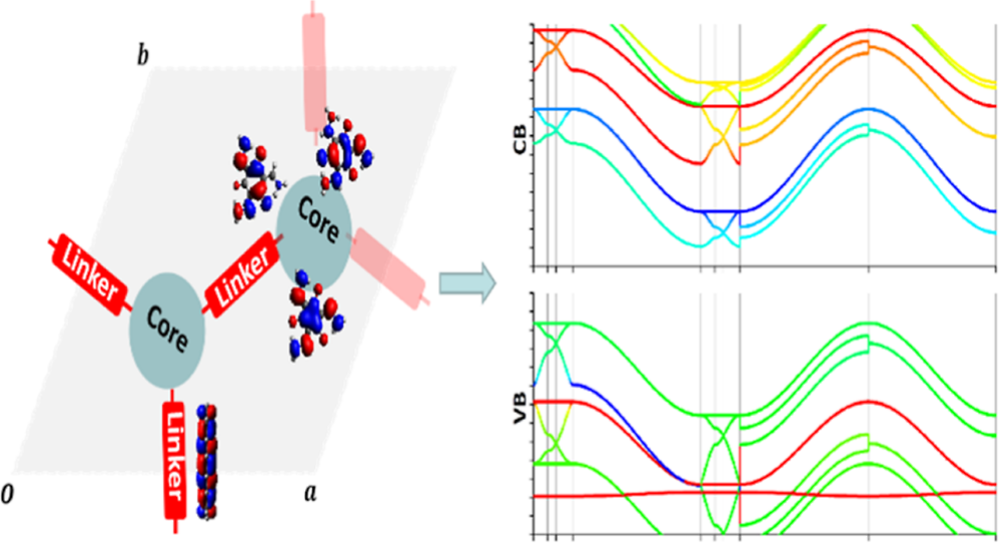

The optical, electronic, and (photo) catalytic properties of covalent organic frameworks (COFs) are largely determined by their electronic structure and, specifically, by their Frontier conduction and valence bands (VBs). In this work, we establish a transparent relationship between the periodic electronic structure of the COFs and the orbital characteristics of their individual molecular building units, a relationship that is challenging to unravel through conventional solid-state calculations. As a demonstration, we applied our method to five COFs with distinct framework topologies. Our approach successfully predicts their first-principles conduction and VBs by expressing them as a linear combination of the Frontier molecular orbitals localized on the COF fragments. We demonstrate that our method allows for the rapid exploration of the impact of chemical modifications on the band structures of COFs, making it highly suitable for further application in the quest to discover new functional materials.

## 1 Introduction

Covalent organic frameworks (COFs) have become one of the most investigated topics in materials chemistry since the first synthesis of COF-1 and COF-5 carried out by Yaghi and co-workers.^1−4^ COFs are constructed from organic molecular building blocks stitched together through dynamic covalent bonds, yielding two- or three-dimensional (two- or three-dimensional) crystalline structures. COFs can be designed in a bottom-up manner from molecular building blocks using reticular chemistry principles.^5^ COF structures can be purposefully targeted by a judicious choice of building blocks, allowing them to be rationally designed and fine-tuned by synthesis to enhance their functionality.^6−9^ For a number of possible applications—such as catalysis and electric conduction—envisioned for COFs, their functional properties are crucially affected by their electronic-structure properties. For example, a narrow band gap of COFs can increase their visible light absorption and may afford an efficient photocatalyst.^10,11^ The development of COF materials for electrochemical energy storage is influenced by the COFs’ ability to transport electronic charges, which is determined by their band structures.^7^ The electronic structure and, by extension, many optoelectronic properties of COFs derive from their topological structures and constituent building blocks, denoted as “cores” and “linkers”.^12^ However, this modular nature of COFs is lost in the commonly adopted approaches to computing COFs’ electronic structures of COFs based on periodic density functional theory (DFT) calculations. Nevertheless, periodic DFT calculations have been routinely employed to study the electronic structures of COFs for functional properties such as charge transport^13−16^ and photocatalysis.^17,18^ However, the high computational costs associated with periodic DFT calculations render them unsuitable for large-scale computational screening of COFs for the desired functions. Moreover, it is complicated to investigate any molecular level factors that impact a COF’s electronic structure and properties within the periodic DFT picture.

A natural step forward in the rationalization of the COF electronic structure is to exploit the modular nature of COFs and connect the electronic properties of a COF’s molecular constituents with the band structure of the framework. Such an intuitive approach will establish a direct connection between the synthetic design approach (based on fragments, connectivity, and topology) and electronic structure calculations (based on the same three elements). The objective of this work is, therefore, to construct a model that computes the band structure of a COF by using the molecular orbitals localized on its constituents and the interactions between them. In essence, the modular approach to COF synthesis will be paired with a modular approach to the interpretation of their electronic properties. Moreover, this modular approach to the computation of COF band structures is computationally efficient, as only DFT calculations of COF fragments are performed.

We first present our methodology and then its application to five prototypical COFs with different framework topologies: **sql**, **hcb**, **kg m**, **dia**, and **pts**, with the first three being 2D topologies and the last two being 3D topologies.^19−26^ We use DFT calculations to extract the parameters of the constituents and their interactions, exploring different partitioning of the COF into fragments. We then express the electronic Hamiltonian in *k*-space and reconstruct the band structure from molecular orbitals on the fragments, which are compared with standard periodic DFT results. Finally, we illustrate how the model derived in this way can be interrogated to explore the effects of chemical modifications to a COF on its electronic structure.

## 2 Methodology

### 2.1 Band Structure from Fragment Molecular Orbitals

We wish to evaluate valence (conduction) bands of the COF by using occupied (unoccupied) Frontier orbitals χ_*a*_ localized on fragments (cores and linkers) of the COF as a basis. The Bloch function ϕ_*a*_ (***k***) of occupied (or unoccupied) local orbitals χ_*a*_ is used as the basis function in ***k***-space
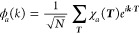
1where ***T*** is the lattice vector with respect to a reference unit cell and χ_*a*_(***T***) is the local orbital χ_*a*_ in the unit cell ***T***.

By neglecting the interaction between occupied and unoccupied orbitals, the valence (or conduction) bands ψ_*i*_ (***k***) of the COF can be expressed as a linear combination of these occupied (or unoccupied) basis functions
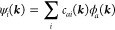
2Here, *c*_*ai*_(***k***) is the linear combination coefficient in ***k***-space and can be calculated by solving the general eigenproblem
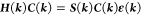
3where [***C***(***k***)]_*ai*_ = *c*_*ai*_(***k***). ***H***(***k***) and ***S***(***k***) is the effective Hamiltonian and overlap matrix in the subspace spanned by the basis function set {ϕ_*a*_ (***k***)}, which can be constructed using real-space Hamiltonian matrices ***H***^(*r*)^ and overlap matrices ***S***^(*r*)^
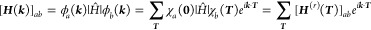
4.1and
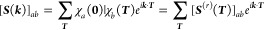
4.2

***H***^(*r*)^ (***T***) (***S***^(*r*)^ (***T***)) is the real-space Hamiltonian (overlap) matrix, whose row and column basis is the local orbital set χ_*a*_ in the reference unit cell and unit cell ***T***, respectively. The band structure of the COF is built from the eigenenergy **ε(***k*) obtained from the reduced model Hamiltonian in eq 3.

**Figure 1:**
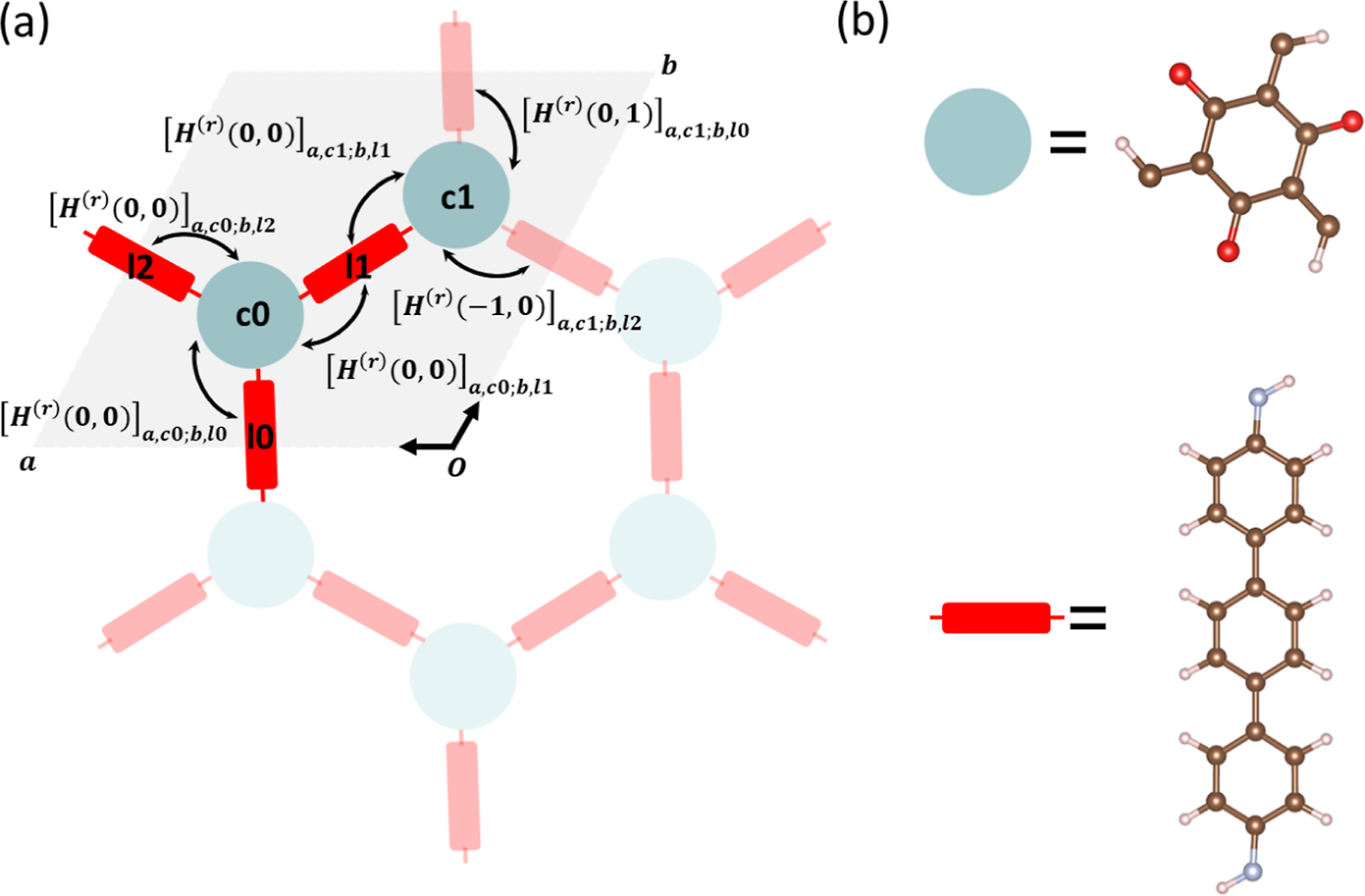
Illustration of the COF model (2D TP-COF as an example). (a) Unit cells of TP-COF and real-space coupling terms between linked core-linker, which are considered in our calculation. The gray area represents the reference unit cell. (b) Chemical structure of core and linker for TP-COF.

In practice, the COF is separated into core and linker fragments, and index *a* can be rewritten as {*a,A*} with *A* being the index for the fragment (*c* for cores and *l* for linkers, as shown in Figure 1). Several MO orbitals on these fragments (saturated with – H) are defined as the local orbital set {χ_*a,A*_}. Then, the matrix elements [***H***^(*r*)^(***T***)]_*a,A;b,B*_ and [***S***^(*r*)^(***T***)]_*a,A;b,B*_ in eqs 4.1 and 4.2 can be obtained using one of three options below,

A. ***T*** = 0, *A* = *B*, and *a* = *b* (orbital energy of the local orbital χ_*a,A*_): [***H***^(*r*)^(***T***)]_*a,A;a,A*_ = ε_*a,A*_ and [***S***^(*r*)^(**0**)]_*a,A;a,A*_ = 1 with ε_*a,A*_ being the DFT orbital energy of χ_*a,A*_.
B. ***T*** = 0, *A* = *B*, and *a* ≠ *b* (orbital coupling between local orbital on the same fragment): [***H***^(*r*)^(***T***)]_*a,A;b,B*_ = 0, [***S***^(*r*)^(***T***)]_*a,A;b,B*_ = 0.
C. Others (coupling terms among different fragments): Only nearest neighbor interactions among fragments are considered. Therefore, if fragment ***A*** in the reference unit and ***B*** in unit cell ***T*** are not in contact by chemical bonding or π–π stacking, [***H***^(*r*)^(***T***)]_*a,A;b,B*_ = 0 and [***S***^(*r*)^(***T***)]_*a,A;b,B*_ = 0.

For these interacting terms, the dimer system of these two fragments is built (adding H atoms if there are bond breaking), and the Fock matrix *F̂* of the dimer is computed using DFT. Notably, in all cases considered here, the relevant fragment orbitals have no weight on the saturating C–H bond, an expected occurrence for pi-conjugated cores and linkers of interest here (a failure of this approach can be easily detected from orbital inspection). Then, the approach to deal with the orbital orthonormalization issue is Löwdin orthogonalization,^27^ which is applied to the subset χ: {χ_*a,A*_ (**0**), χ_*b,B*_ (***T***)} in the dimer system
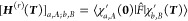
5with
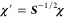
6

Here, ***S*** is the overlap matrix of set **χ**. Then [***S***^(*r*)^(***T***)]_*a,A;b,B*_ = 0.

In essence, the band calculation requires the computation of isolated fragments and the dimer of interacting fragments in all possible orientations. These calculations require just a few hours on a single CPU. The results are somewhat dependent on the choice of the orbitals to include, but as discussed below, it is possible to establish a protocol that yields good results most of the time.

### 2.2 Computational Details

In order to assess the reliability of the above parametrized theory model, electronic band structures of five different COFs with the **sql**, **hcb**, **kg m**, **dia**, or **pts** topology were determined by both the model proposed here and the standard periodic DFT. The five COFs are COF-366-Zn,^20^ COF-701,^21^ TpPa-1,^22^ TP-COF,^23^ dual-pore COF,^24^ COF-300,^25^ and 3D-py-COF.^26^ All geometry optimizations and band structure calculations of the periodic structures of the COFs were performed at the DFT level with the projector-augmented wave method using the Vienna ab initio simulation package (VASP).^28,29^ The exchange and correlation potentials were treated with the generalized gradient approximation (GGA) functional of the Perdew, Burke, and Ernzerhof (PBE) functional (dispersion interactions were taken into account using DFT-D3). Geometry optimizations and electronic band structures were calculated at the same level of theory. A kinetic-energy cutoff of 450 eV was used to define the plane-wave basis set. Tolerances of 1 × 10^–5^ eV and 0.01 eV/AÅ were applied during the optimization of the Kohn–Sham wave functions and the geometry optimizations, respectively.

For fragment calculations of carved-out cores and linkers, the B3LYP functional and the 6-31g(d) basis set were used to obtain the information on local FMOs of fragments using the Gaussian 16 package.^30^ To construct the band structures of a COF, the Frontier molecular orbitals and their immediate next orbitals on the core/linker were used. For instance, the HOMO and HOMO – 1 of the cores and the HOMO of the linkers in the reference unit cell were used for valence band (VB) structure calculation. In addition, by adding more FMOs per core or linker, the lowest numbers of FMOs *N*_orb_ (per core/linker), core-linker couplings (*N*_cl_), and π–π couplings (*N*_π_) were eventually obtained from the fragment numbers (*N*_c_ for core and *N*_l_ for linker) in the unit cell. The parameter set (*N*_orb_, *N*_cl_, *N*_π_, *N*_c_, and *N*_l_) can be readily used to construct band structures for other COFs with the same framework topology.

## 3 Results and Discussion

### 3.1 Electronic Structure of the Topological Structure of **hcb** Net

**Figure 2:**
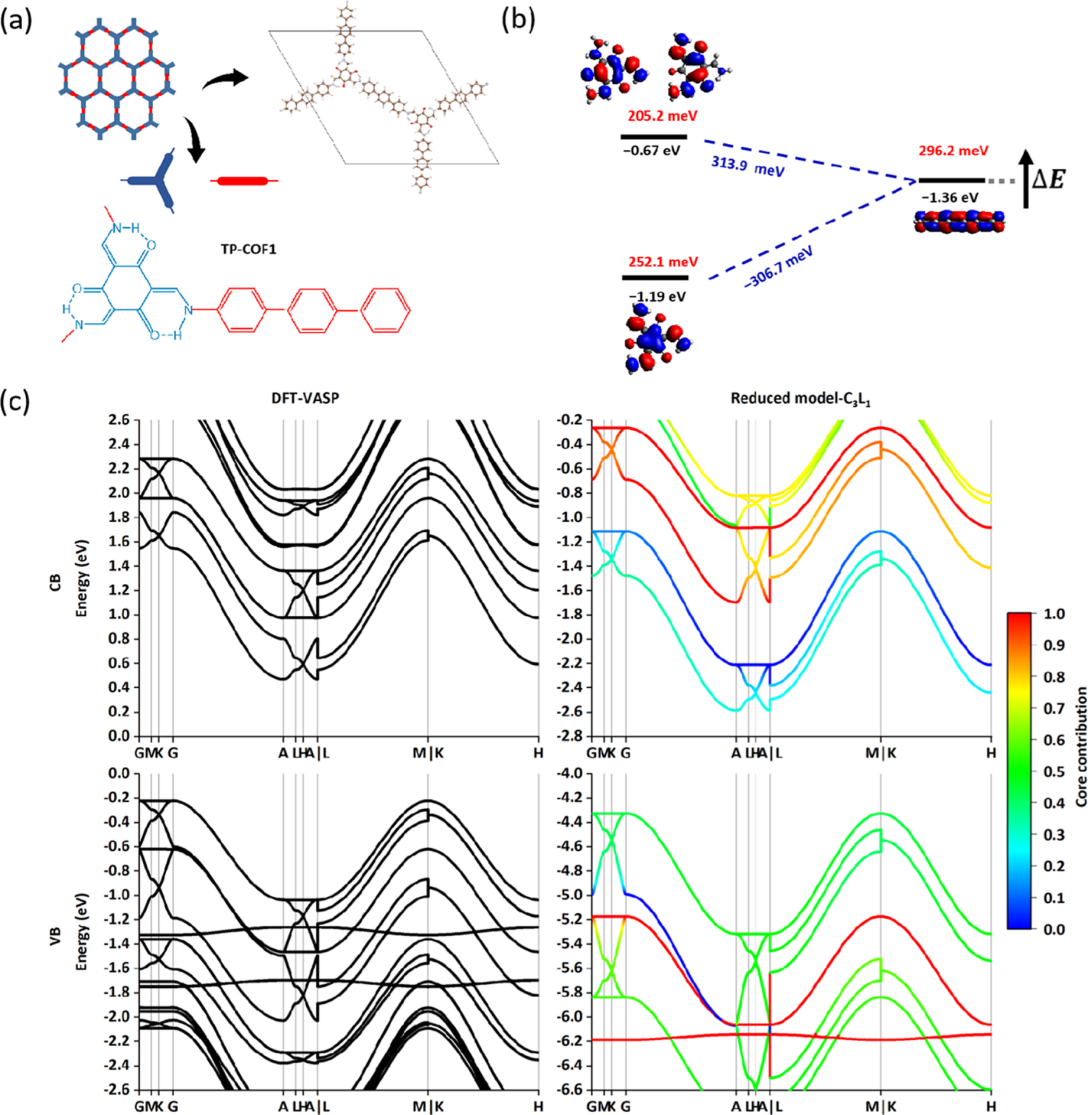
Results of TP-COF (**hcb**). (a) Topological structure with the partitioning core and linker. (b) Parameter set for CBs (orbital energy terms in black, core-linker coupling terms in blue, and π–π coupling terms on *c* direction in red). (c) CBs and VBs band structure results. The color in the right computed band diagram (the reduced model) encodes the fractional contribution of the core orbital on the corresponding band. In Section 3.3, we explored the effect of shifting the energy of the linker’s LUMO by a quantity Δ*E*.

As shown in Figure 2a, the **hcb** net of COFs contains a triangle vertex figure, and hence, all of the angles are 120°, depicted by one core connecting with three linkers. Therefore, the least number of cores and linkers that supply the molecular orbitals in the above methodology should be 2 and 3 in one unit cell. It is important to confirm the minimal orbital set and corresponding minimal parameter set of **hcb** COF (i.e., orbital energies of cores and linkers, couplings between linked core-linker orbitals, and π–π stacking core–core/linker–linker orbitals) to construct a reasonably accurate reduced model. However, despite the same **hcb** net, there are still various COFs reported by previous works, showing unique structure properties or different compositions,^21−23^ such as TpPa-1 and TP-COF, differing by the linkers *p*-phenylenediamine and 4,4-diamino-*p*-terphenyl, respectively. As shown in Figure 2b, two nearly degenerated FMOs of TP-COF (also see the Frontier orbital energies in Table S1 in the Supporting Information) can be found from the core, which are HOMO and HOMO – 1 for VBs or LUMO + 1 and LUMO + 2 for conduction bands (CBs). Therefore, three FMOs from the core (HOMO, HOMO – 1, and HOMO – 2 for VBs or LUMO, LUMO + 1, and LUMO + 2 for CBs) and 1 FMO from the linker (HOMO for VBs or LUMO for CBs) are needed to reproduce the Frontier band structure, and the parameter set is denoted as C_3_L_1_. Correspondingly, 6 core orbital energy terms, 3 linker orbital energy terms, 18 linked core-linker coupling terms, 18 core–core π–π coupling terms, and 3 linker–linker π–π coupling terms are considered to construct the minimal parameter set and calculate the Frontier band structure. The Frontier band structures of CBs and VBs are illustrated and compared to VASP results in Figure 2c. The general band structure results, including the bandwidth of conduction and VBs of C_3_L_1_, are comparable to the VASP results, suggesting that the approximate methodology is promising. Note that VASP calculations set the zero of the energy to the Fermi level and that the band gap is smaller for VASP because of the choice of the GGA functional. The bands computed for the C_3_L_1_ model are also projected on the component basis set and color-coded in Figure 2c according to the weight of the orbital in the core. The core and linker dominate VBs and CBs, respectively, with the states near the band gap edge comparatively more mixed. A model computed with a reduced number of orbitals (C_2_L_1_), reported in Figure S1a,b, displays a poorer agreement with the full calculation, highlighting the importance of the additional orbital on the core for the description of the relevant states. For comparison, the Frontier band structures of CBs and VBs based on the nonhybrid functional (PBE) are calculated and shown in Figure S1e,f, proving the significant exchange in the orbital localization for the present approach, which is likely to impact the interfragment coupling.^31−34^

Similar results are obtained for the topologically analogue TpPa-1 (**hcb**), which has the same core but the short linker with TP-COF, where good quality CB and VB band can be obtained from a reduced model of similar size (C_3_L_1_) (see Figure S2). Thus, the simplest set of **hcb** COFs should be 2/1 orbital per core/link with eight parameters: three orbital energies, two bond coupling terms, and two/one π–π coupling terms for core/linker. The two cases **hcb** COFs considered here display small band gap and large band dispersion (small effective masses for both holes and electrons^13^), suggesting that they are suitable for applications requiring good charge transport. Indeed, some other COFs like Py-COF^35^ and PTM-CORF^36^ belonging to **hcb** net have been widely studied because of their electronic structure favorable to charge transport. As discussed by Rico Gutzler,^37^ the strong hybridization between adjacent monomers leads to strong orbital splitting, which is manifested by the strong mix of Frontier orbitals from core and linkers (marked in green in Figure 2c) and the separated energy lines with large energy dispersion. It should be noted that alternative partitions between “core” and “linkers” are possibly leading to somewhat different results, which will be discussed at the end of Section 3.2 for all of the different topological COFs.

### 3.2 Example Results of the Other Four COF Topologies

**Figure 3:**
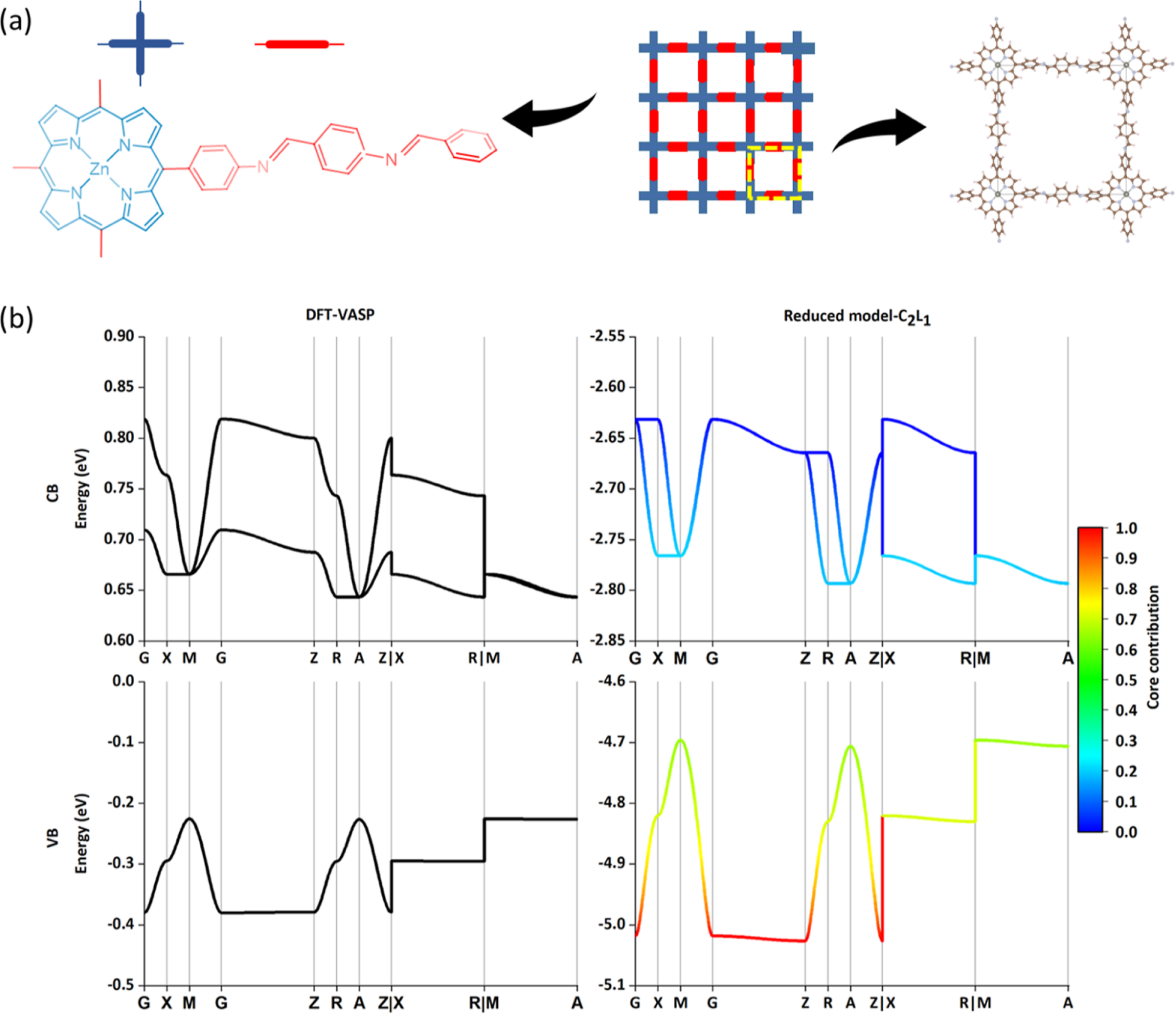
Results of the COF-366-Zn (**sql**). (a) Topological structure with the partitioning core and linker. (b) CBs and VBs band structure results via DFT and reduced model with C_2_L_1_. The color in the band diagram has the same meaning as Figure 2.

From Figure 3a, COFs with an **sql** topological structure usually exhibit square arrangements with a 90° angle between linkers.^38^ One core (cross symbol in blue) connects with four linkers (line symbol in red), which makes **sql** COFs 2D layered crystalline. Therefore, the parameters (orbital energies and the couplings) are derived from one core and two linkers in the primitive cell. A classic example of **sql** COF is COF-366-Zn, which is prepared by 5, 10, 15, 20-tetrakis(4-aminophenyl)-zincporphinato [Zn(TAP)] with 1,4-benzene-dicarboxaldehyde, as shown in Figure 3a.^20^ The quasi-degeneracy of LUMO + 1 and LUMO, as well as HOMO–1 and HOMO in the core fragment (shown in Figure 3a and Tables S2) means that it requires at least two orbitals for either conduction or VB. The minimal model C_2_L_1_ produces CB and VB in COF-366-Zn (**sql**) in good agreement with VASP calculations, as seen in Figure 3b. On the other hand, if more FMOs like HOMO – 2 from the core and HOMO – 1 from the linker are added to the model, a large number of parameters needs to be evaluated, while there is no distinct improvement in the top VBs and the bottom CBs, those determining the key electronic properties of the materials.^39^ From this additional example, it seems the inclusion of the smallest number of (quasi) degenerate orbitals from the core and linker is sufficient to produce useful reduced models. Considering that COF-366-Zn is a prototype of **sql** COF with porphyrin core and linear links, we may argue that the minimal model for in **sql** COFs would include five parameters, two orbital energies from C_2_L_1_, one bond coupling term, and one/one π–π coupling terms for core/linker.

**Figure 4:**
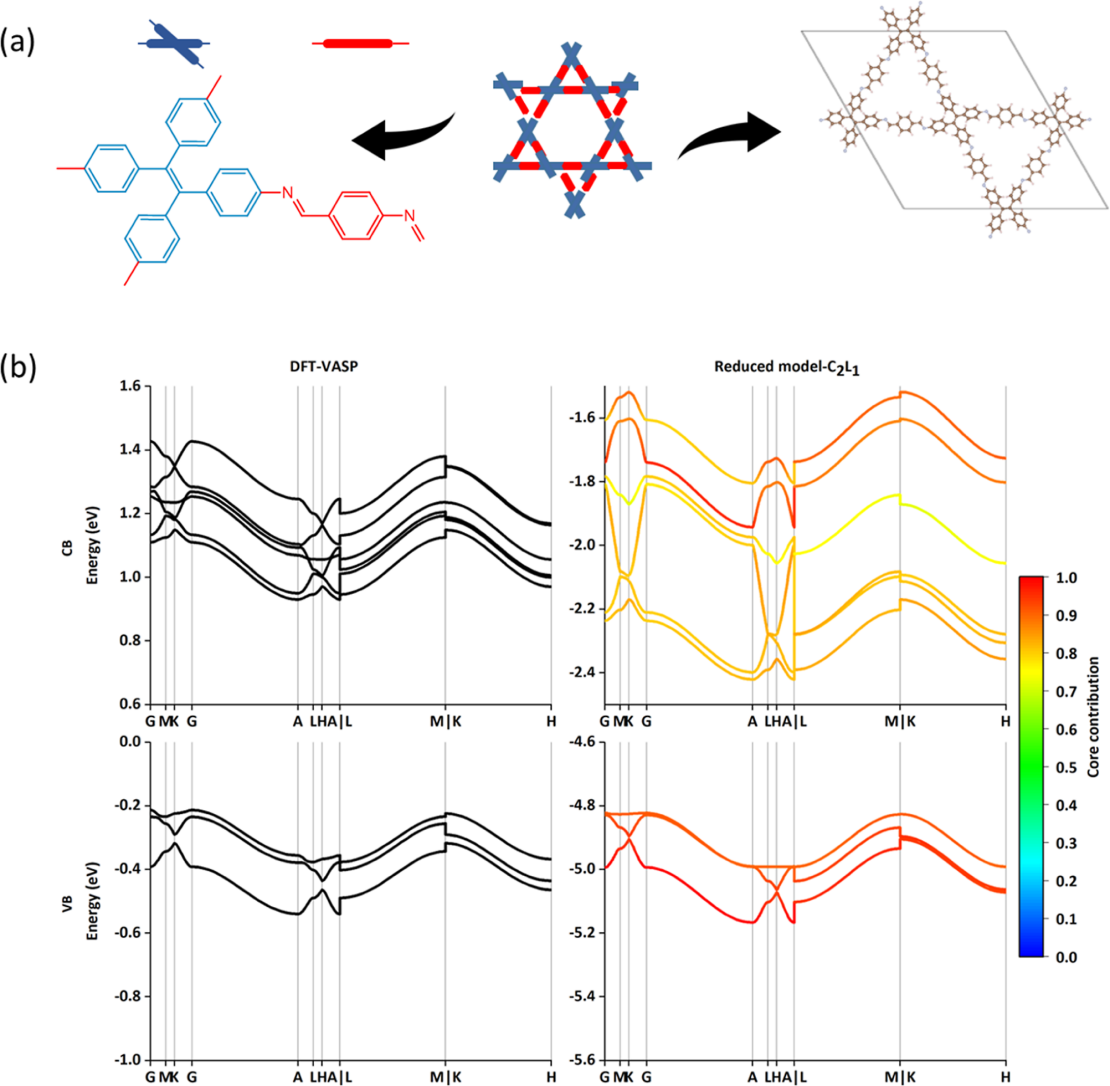
Results of dual-pore COF (**kg m**). (a) Topological structure with the partitioning core and linker. (b) The CBs and VBs band structure results via DFT and reduced model with C_2_L_1_.

The **kg m** COFs display some similarities with **sql** materials, they are 2D layered materials and the core is connected to four linkers but is the angle between the linkers is 60 and 120° for **kg m**, as shown in Figure 4a, unlike **sql** where it is 90°. Therefore, a minimum parameter set was set to calculate the electronic band structure derived from three cores and six linkers in the primitive cell and couplings from the topological properties of **kg m**. Here, we take dual-pore COF as an example, which consists of 4,4′,4″,4‴-(ethene-1,1,2,2-tetrayl)-tetraaniline and terephthalaldehyde.^24^ Based on the partition in Figure 4a, the orbital energies of FMOs are found in Table S3, and hence, two and one FMOs from the core (red) and linker (blue) are considered here. As illustrated in Figure 4b, the present partitioning with parameter C_2_L_1_ is adequate to reproduce the CB and VB from VASP. Similar to the above COFs discussed earlier (**sql** and **hcb**), the results are not improved by including additional orbitals to the models, as shown in Figures S4a,b.

**Figure 5:**
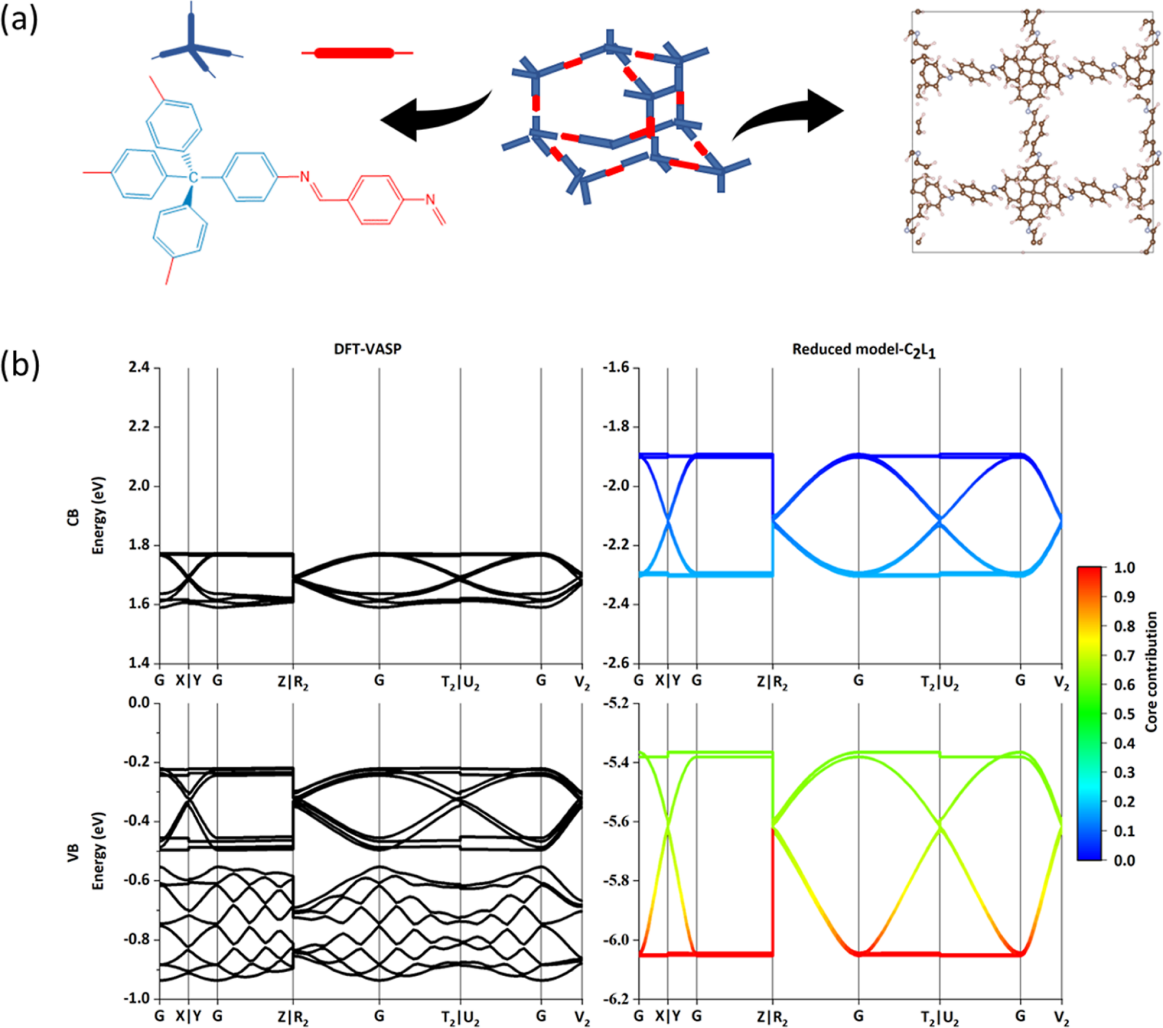
Results of COF-300 (**dia**). (a) Topological structure with the partitioning fragment core and linker. (b) CBs and VBs band structure results via DFT and reduced model with C_2_L_1_.

Unlike the other nets seen so far, the covalent framework in **dia** extends in 3D, and the core-linker connection is similar to that of diamond, where the cores play the role of tetrahedrally coordinated C atoms, while the linkers act as the bonds. In Figure 5, the core and linker numbers are 4 and 8 in the primitive cell, respectively. As an example of this class, we considered COF-300 synthesized by the condensation of tetrahedral building block tetra-(4-anilyl) methane and linear linker unit terephthaldehyde,^25^ and we illustrated our partitioning (core in red and linker in blue) in Figure 5a. Determined from Table S4, parameter C_2_L_1_ is adopted to calculate the Frontier bands (CB and VB), which are well fitted with that from VASP results in Figure 5b. Also in this case, the addition of extra orbitals like HOMO – 2 or LUMO + 1 does not modify the bottom and top of CB and VB but it can modify the shape of other bands (see Figure S5a,b). It should be noted that while the band shape is similar when computed from the minimal model or VASP, the minimal model displays a larger dispersion, although both models agree on the very large electron and hole effective mass (flat bands). If one wishes to generalize the approach to other **dia** COFs, the minimal model would include three orbital energies from C_2_L_1_ and two bond coupling terms.

**Figure 6:**
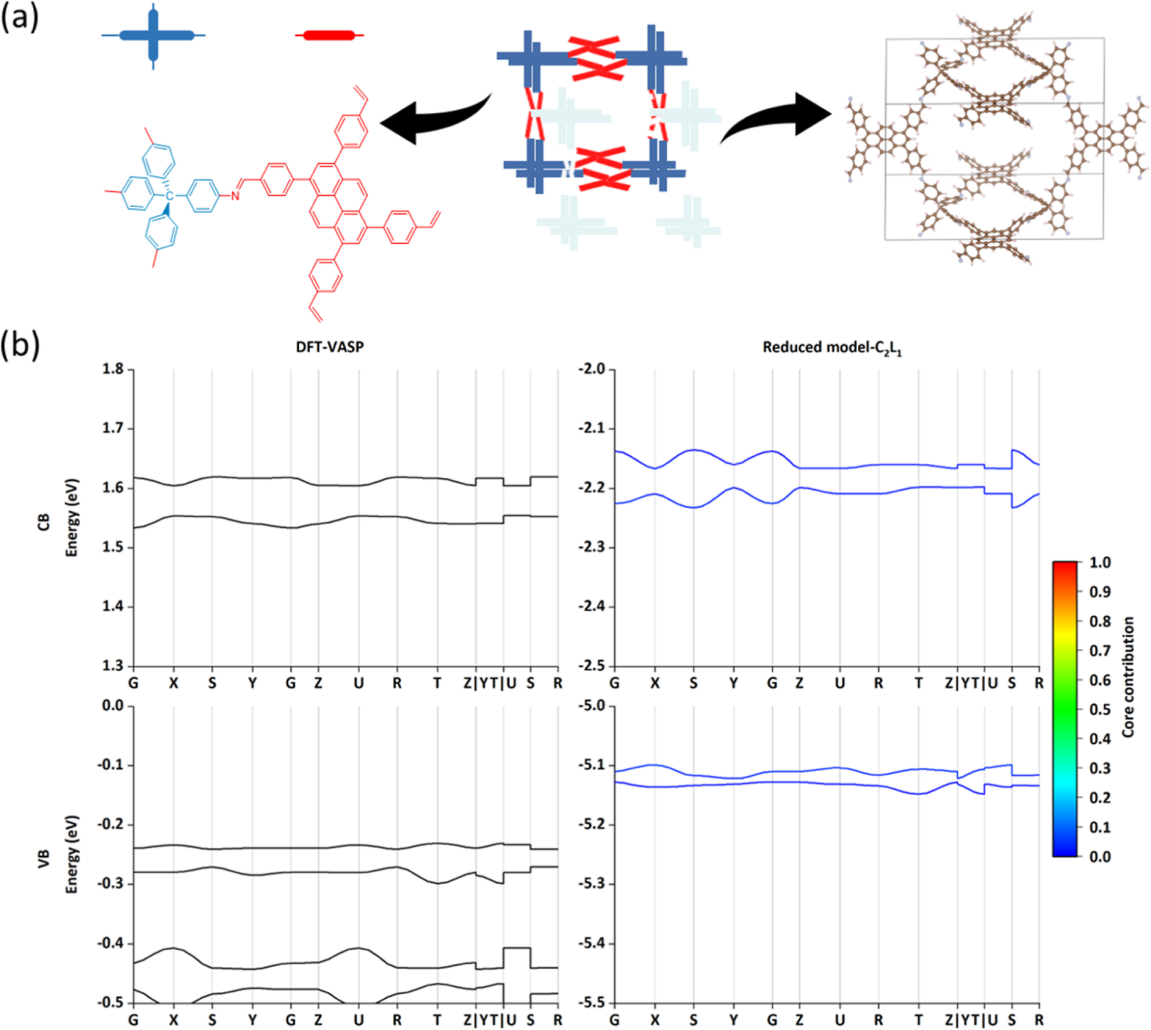
Results of the 3D-Py-COF (**pts**). (a) Topological structure with the partitioning fragment core and linker. (b) CBs and VBs structure results via DFT and reduced model with C_2_L_1_.

According to the previous examples of COF-366-Zn, TpPa-1, TP-COF, dual-pore COF, and COF-300, all the obtained band structures show relatively broad bands. When it comes to 3D-Py-COF, the property of the band structures is changed. As shown in Figure 6a, 3D-Py-COF as a typical **pts** net shows a 2-fold interpenetrated network with square or tetrahedron vertex figure in Figure 6a,^26^ and the obtained CBs and VBs from both VASP and the present approach are almost flat lines in Figure 6b, meaning the large effective mass of electron and hole denotes the low conductivity and higher transparency of COFs. The origin of this feature can be ascribed to the orbital energies (shown in Table S5) since the large energy difference between orbitals in the covalently connected fragment is very large, causing poor mixing and negligible dispersion.

The reduced model correctly reproduces the flat bands, but it is difficult to draw a direct comparison with the results of VASP in this situation [the results are similar if more orbitals are included in the reduced model (Figure S6)]. It should be noted that in the presence of dispersionless band in a very large unit cell, we expect the charge carrier to become localized by the effect of electron–phonon coupling^40^ and the details of the band becomes irrelevant. In this sense, the reduced model retains its usefulness as it is able to identify these cases correctly, while a more quantitative agreement is found for bands with large dispersion. From this point of view, the chemical composition is very important to the properties of band structures of COFs, and it can modify the conductivity effectively. This is also the reason why COF-701 has the same topological structure (**hcb**) of TpPa-1 and TP-COF, but the VBs from the reduced model (see Figure S7) are flatter than that of the other two, also in this case because of the large mismatch between orbital energies of the fragments (see Table S6).

From all the examples above, the partitioning core and linkers from different topological COFs all reproduce the Frontier bands (CBs and VBs) from the VASP calculation. It is clearly possible to devise alternative partitions between core and linker, which often lead to poorer results. For example, the band structure of TP-COF (**hcb**), COF-366-Zn (**sql**), dual-pore COF (**kg m**), 3D-py-COF (**dia**), and COF-300 (**pts**) by the alternative partitioning are shown in Figures S1c,d and S3–S6c,d. However, as discussed more extensively in the Supporting Information, it is possible to devise a general approach to identify the most effective partitioning between core and linker entirely based on dimer calculations and not requiring expensive VASP calculations. The best partitioning is the one for which one observe the best localization of HOMO and LUMO of the dimer in the respective fragment and this is illustrated for all systems considered in Figures S8–S13 and Table S7.

In essence, the practicability and accuracy of the theoretical model have been effectively proved for a sample of COFs of the **sql**, **hcb**, **kg m**, **dia**, and **pts** families. When considering the computational costs of the full electronic structure calculation (shown in Table S8), we observe that the present approach can provide the most valuable bands (Frontier CBs and VBs) about 2 orders of magnitude more rapidly using the simplest parameters (Table 1). Here, *N*_orb_ (m/n) are the numbers of selected Frontier orbitals (such as HOMO, HOMO – 1, etc., and LUMO, LUMO + 1, etc.) per core/linker, which represent the least numbers of FMOs needed to calculate the band structures of different topological COFs. As we discussed for sql, hcb, and kgb (with data shown in the Supporting Information), there is no change in the band structure when more orbitals can be included. It is worth noticing that there is no need of the VASP reference to establish a convergence of the band structure as the number of considered orbitals are increases. However, it seems that including a single Frontier orbital (two if degenerate) for each linker and core is sufficient for most cases. When comparing the COF-300 and 3D-Py-COF, the same core with different linkers can form distinct topological structures like **dia** and **pts**; hence, their band structures are entirely different. By contrast, TpPa-1 and TP-COF also have the same core but belong to the same topological net **hcb**, exhibiting a similar band structure. Therefore, the band is determined mostly by the topology (similar topology tends to give a similar band structure) and not so much by the constituents (one can have similar constituents and obtain a different band structure). The proposed method provides a very clear framework to rationalize these patterns.

**Table 1:** Topological Structures, Fragment Numbers (*N*_c_ for Core and *N*_l_ for Linker) in Unit Cells, FMO Numbers *N*_orb_ (per Core/Linker), Core-Linker Couplings (*N*_cl_), and π–π Couplings (*N*_π_) for Different COF Samples

topology		*N*_c_	*N*_l_	*N*_orb_	*N*_cl_	*N*_π_	example
**hcb**	2D	2	3	3/1	18	21	TpPa-1, TP-COF
		4	6	2/1	24	22	COF-701
**sql**		1	2	2/1	4	4	COF-366-Zn
**kg m**		3	6	2/1	36	18	dual-pore COF
**dia**	3D	4	8	2/1	64	0	COF-300
**pts**		2	2	2/1	8	0	3D-Py-COF

A possible advantage of the localized representation of band orbital for COF is the possibility to consider multiple transport mechanisms once a charge carrier (hole or electron) is added to the material^41,42^ The additional parameter to consider is the local electron–phonon coupling or charge reorganization energy, which measures the stabilization of the additional charge caused by the relaxation of the nuclei. This quantity, typically in the range between 0.1 and 0.3 eV for fragments of this size, can be readily computed for the individual fragments.^43^ If the reorganization energy is larger than the electronic bandwidth, the carrier will become localized and the transport will take place as a series of hopping events between these states. In the opposite limit of much larger bandwidth a common assumption is that the transport is “band-like”, i.e., characterized by delocalized carriers scattered by impurities.^44^ Our results already indicate that both limits can be encountered in different COFs. An additional complication is that COFs are relatively “soft” materials, and even when they satisfy the criteria for band transport, they can display the characteristics of *transient localization*, where the dynamics of the COF at room temperature localizes the carrier and limits the transport.^42,44^ Further work is required to fully characterize these regimes, but in all cases, they require a reduced model of the electronic structure as a starting point.

### 3.3 Effect of Changing Individual Parameters

As shown in Table 1, VBs (CBs) can be described by only several occupied (unoccupied) Frontier orbitals of local core and linker fragments. For a fixed topology, the band depends only on a limited number of parameters (orbital energies and coupling), which can be controlled to a certain extent via chemical modifications (sufficiently small not to change the topology). Building a minimal model based on the smallest set of parameters provides an intuitive and predictive model of how certain modifications change the electronic band and can be used to optimize the COF for certain properties, e.g., larger band gap (for transparency) or larger band dispersion (for higher mobility).

**Figure 7:**
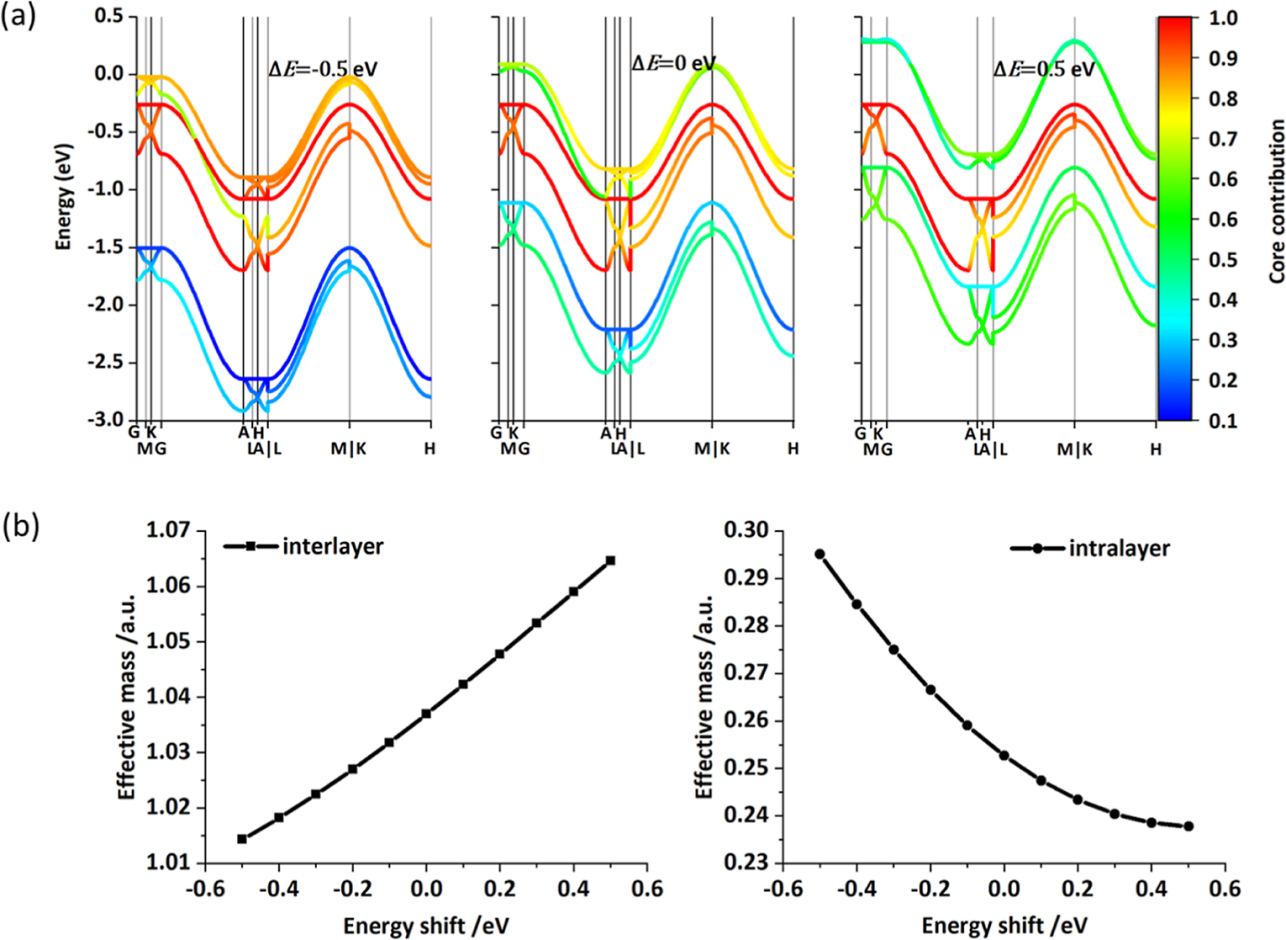
(a) Projected band structure results for CBs of TP-COF using the model with the energy shift of the linker LUMO energy. Color presents the contribution of core orbitals in bands. (b) Effective mass for interlayer (left, A[0.0, 0.0, 1/2] → Γ[0.0, 0.0, 0.0] k path) and intralayer (right, A[0.0, 0.0, 1/2] → H[1/3, 1/3, 1/2] k path) electronic transport with energy shift of the linker LUMO energy (original: −1.36 eV in Figure 2b).

To explore this possibility in some detail, we considered **hcb** COFs, where, as we have seen, in practice, one orbital of the linker is enough, while more orbitals of the core are required because at least three orbitals of the core are needed. Despite this, the symmetry-protected degenerate orbitals can be regarded as one orbital by introducing a phase factor [see Figure S14, where cos θ/sin θ are orbital coefficients (not normalized) based on the Hückel theory] and the number of parameters of the model can be further reduced. Taking TP-COF as an example, only three energy (or two energy differences if we only focus on the band dispersion), two intralayer core-linker couplings, and three interlayer π–π couplings are needed to reproduce the band structure, as shown in Figure 2. A reduced model like the one depicted in Figure 2b can be used to evaluate the hypothetical effect on the band structure of altering some of the parameters by chemical modification. The easiest modification that can leave the COF structure largely unchanged is the addition of chemical substituents that shift the orbital energy levels in a controllable way. To exemplify how the current model can be used in practice, we have recomputed the CB of TP-COF, dominated by the linker, by shifting the energy of the LUMO on the linker by Δ*E* (as shown in Figure 7a). Going from negative to positive Δ*E*, the band edge moves to higher energy, and the composition of the orbital evolves from being completely localized on the linker to being fairly delocalized between linkers and core. Furthermore, the relation between these parameters and the effective mass for interlayer and intralayer electron transport can also be computed and is reported in Figure 7b. The Supporting Information explores further the effect of changing the coupling terms, which modulate the bandwidth, and the corresponding effect on the effective mass (Figures S15–S17).

A reduced model of this type can therefore become a powerful tool for the design of new COFs with fine-tuned electronic properties. For this, the primary application of the method as we envisaged is to help the user build a reduced model for a given topology and then ask the question of what would happen to a range of modifications of the structure (shifting energy levels via chemical substituents, strengthening coupling between cores by planarizing linkers, etc.). If the model is validated for a reference structure, it is possible to assume that the model will be able to predict the effect of certain modifications, which is a valuable tool for the design of the new COFs.

## 4 Conclusions

The present approach based on a reduced model Hamiltonian calculation has proven efficient in identifying a transparent and intuitive relation between the orbital properties of the cores/linkers and the electronic structure of COFs. The model has been applied to examples from five common topological COFs (**sql**, **hcb**, **kg m**, **dia**, and **pts**) and it has been shown that a minimal model based on very few parameters can describe the top valence and bottom CBs. The parameters of the model, including the selection of the orbitals to include and the best partitioning of the COF into core and linker fragments, can be determined from simple calculations of fragments and dimers. The method has the advantage of being very rapid and potentially suitable for new functional materials discovering and supplying the basic role for the future virtual screening of COFs. The main advantage, however, is the ability to provide an intuitive relation between the COF components which can be tailored by synthesis (energy levels and strength of the coupling), providing a simple approach to explore the effect of chemical modifications on the electronic properties. In this way, the modular approach to COF synthesis will be paired with a modular approach to the interpretation of the electronic properties.

## Supporting Information Available

Molecular orbitals information on the partitioning fragments for the studied COFs; comparison of the present partition with the alternative one; consumption of cup-time (hour) of the whole and Frontier bands from VASP and optimal parametrized theory model; comparison of CBs and VBs for different orbitals involved and partitioning; geometrical structure and orbitals properties of dimer of connected fragments; orbitals illustration of four-member ring and three-member ring systems; and projected band structure results for CBs of TP-COF by parameters shift (PDF)
