# Supplementary material for: Intuitive and Efficient Approach to Determine the Band Structure of Covalent Organic Frameworks from Their Chemical Constituents
Source: J Chem Theory Comput. 2024 Feb 2;20(3):1252–62. doi: 10.1021/acs.jctc.3c01302 (PMC10867833; doi:10.1021/acs.jctc.3c01302)
Supplement: Supplementary file 1 — ct3c01302_si_001.pdf [file ct3c01302_si_001.pdf]

An Intuitive and Efficient Approach to Determine the Band Structure of Covalent Organic Frameworks from Their Chemical Constituents

Changchun Ding[ǂa,b]*, Xiaoyu Xie[ǂb], Linjiang Chen[c], Alessandro Troisi[b]*

[a] *School of Science, Xihua University, Chengdu 610039, China*

[b] *Department of Chemistry, University of Liverpool, Liverpool L69 3BX, United Kingdom*

[c] *School of Chemistry and School of Computer Science, University of Birmingham, Birmingham B15 2TT, UK*.

*E-mails: dingcc@liverpool.ac.uk, A.Troisi@liverpool.ac.uk

Table S1. The molecular orbitals information of the partitioning fragments with the good band fitting in TP-COF

Cutting of TP-COF	Orbital	Energy/eV
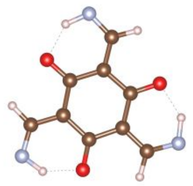 Core	LUMO+3	1.7557
	LUMO+2	-0.6701
	LUMO+1	-0.6702
	LUMO	-1.1910
	HOMO	-5.6169
	HOMO-1	-5.6170
	HOMO-2	-6.1641
	HOMO-3	-6.3154
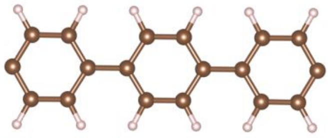 Linker	LUMO+2	0.0247
	LUMO+1	-0.2889
	LUMO	-1.3565
	HOMO	-5.5297
	HOMO-1	-6.7106
	HOMO-2	-6.7621

Table S2. The molecular orbitals information of the partitioning fragments with the good band fitting in COF-366-Zn

Cutting of COF-366-Zn	Orbital	Energy/eV
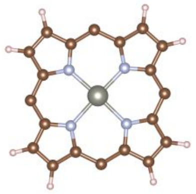 Core	LUMO+2	-0.7932
	LUMO+1	-2.2605
	LUMO	-2.2608
	HOMO	-5.0220
	HOMO-1	-5.4021
	HOMO-2	-6.4175
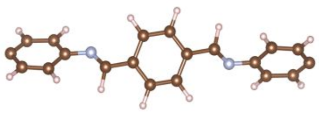 Linker	LUMO+2	-0.1864
	LUMO+1	-1.6891
	LUMO	-2.6478
	HOMO	-5.3263
	HOMO-1	-5.7238
	HOMO-2	-6.6331

Table S3. The molecular orbitals information of the partitioning fragments with the good band fitting in dual-pore COF

Cutting of dual-pore COF	Orbital	Energy/eV
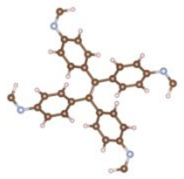 Core	LUMO+2	-1.2130
	LUMO+1	-1.2542
	LUMO	-1.8412
	HOMO	-5.0800
	HOMO-1	-6.1403
	HOMO-2	-6.2439
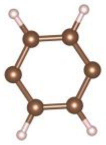 Link	LUMO+2	2.5019
	LUMO+1	0.1610
	LUMO	-0.0214
	HOMO	-6.6185
	HOMO-1	-6.7470
	HOMO-2	-9.1133

Table S4. The molecular orbitals information of the partitioning fragments with the good band fitting in COF-300.

Cutting of COF-300	Orbital	Energy/eV
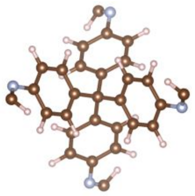 Core	LUMO+2	-1.0758
	LUMO+1	-1.3737
	LUMO	-1.4093
	HOMO	-5.7928
	HOMO-1	-5.8099
	HOMO-2	-6.2327
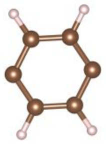 Linker	LUMO+2	2.4997
	LUMO+1	0.1544
	LUMO	-0.0217
	HOMO	-6.6192
	HOMO-1	-6.7432
	HOMO-2	-9.1177

Table S5. The molecular orbitals information of the partitioning fragments with the good band fitting in 3D-py-COF.

Cutting of 3D-py-COF	Orbital	Energy/eV
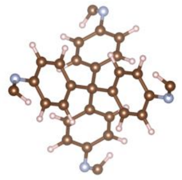 Core	LUMO+2	-1.0070
	LUMO+1	-1.2824
	LUMO	-1.3907
	HOMO	-5.7230
	HOMO-1	-5.7796
	HOMO-2	-6.2027
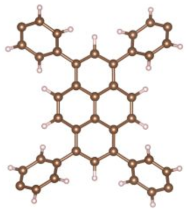 Linker	LUMO+2	-0.4794
	LUMO+1	-0.7440
	LUMO	-1.7871
	HOMO	-4.9487
	HOMO-1	-5.9947
	HOMO-2	-6.4174

Table S6. The molecular orbitals information of the partitioning fragments with the good band fitting in COF-701

Cutting of COF-701	Orbital	Energy/eV
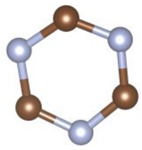 Core	LUMO+2	2.7110
	LUMO+1	-1.6372
	LUMO	-1.6458
	HOMO	-7.4838
	HOMO-1	-7.5010
	HOMO-2	-9.0742
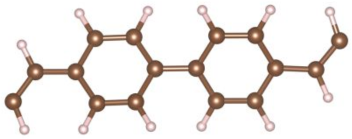 Linker	LUMO+2	-0.1368
	LUMO+1	-0.5038
	LUMO	-1.4958
	HOMO	-5.4986
	HOMO-1	-6.4282
	HOMO-2	-6.8446

Table S7. The comparison of the present partition with the alternative one according to the bond length from partitioning site and the localization of HOMO and LUMO in each portion (core and link).

Comparison	TpPa-1	TP-COF	COF-366	dual-pore	COF-300	3D-py-COF
Bond length	√	√	√	√	×	×
HOMO	–	√	–	√	–	√
LUMO	√	–	√	–	√	√
Combined	√	√	√	√	√	√

The partitioning between fragments may influence the results and often the best choice is not completely intuitive from the chemical diagram. In the main manuscript we report the results with the closest match with the VASP calculations while in the SI we report alternative partitions. In this table, the best partition for COF-366-Zn, TpPa-1, TP-COF, dual-pore COF, COF-300 and 3D-py-COF presented all are determined by the combination of the localization properties of HOMO and LUMO in dimer rather than the bond distance in the partitioning sites. As powerfully illustrated in **Figs. S8-S13**, the best partitioning marked in red color effectively separates the frontiers orbitals aside while the blue one almost equally divide these orbitals. Therefore, it is possible to determine the best partition by considering the frontiers orbitals of a dimer composed by the two connected fragments, that is the best partitions have HOMO and LUMO more localized on each of the two portions, such as core and linker in **Figs. 1-6** and **Figs. S8-S13** (red one)

Table S8. The consumption of cup-time (hour) of the whole and frontier bands from VASP and optimal parameterized theory model.

Time	COF-366	COF-701	TpPa-1	TP-COF	dual-pore	COF-300	3D-py-COF
VASP	64.5	64.4	17.4	97.6	131.1	157.3	199.5
Gaussian	0.42	0.32	0.09	0.23	0.71	0.87	1.11

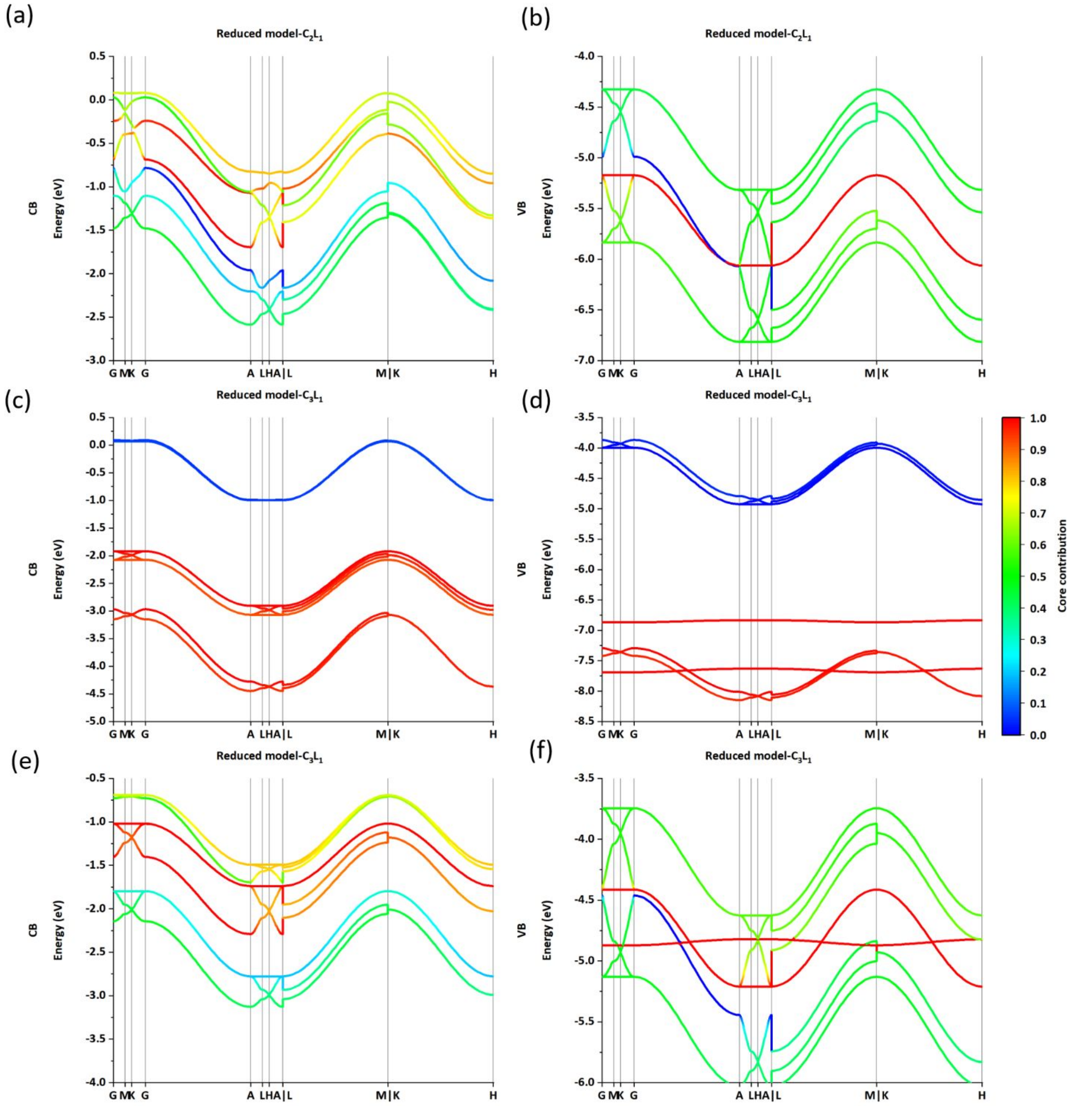

Figure S1. The conduction and valence bands of TP-COF. The CB (a) and VB (b) with the reduced parameters of C_2L_1 from **Fig. 2**; the CB (c) and VB (d) with the parameters of C_3L_1 from another partition from **Fig. S8** (blue one); the CB (e) and VB (f) determined from the PBE functional in Gaussian 16.

(a)

(b)

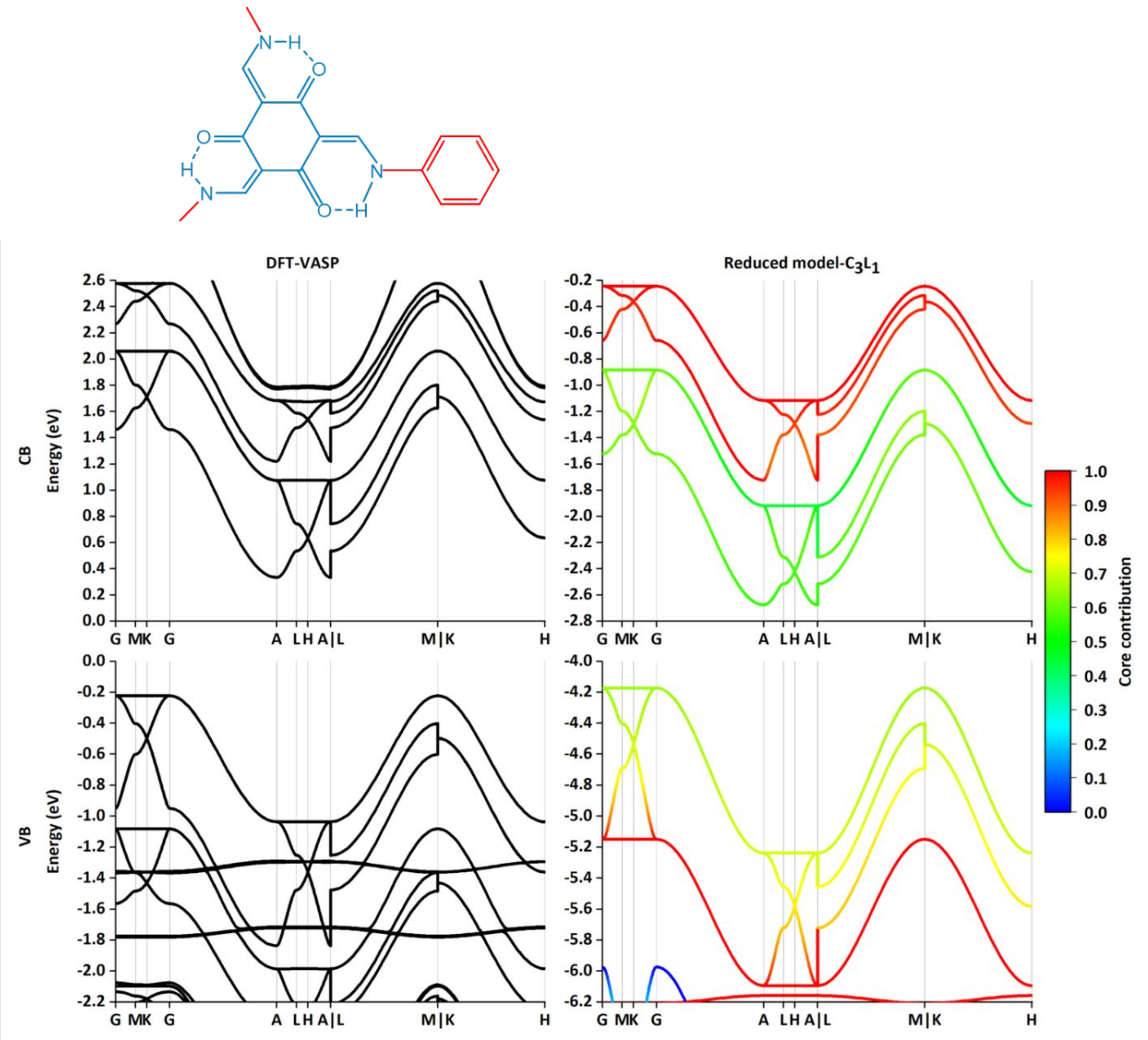

Figure S2. Topological structure with the partitioning core and linker (a) and calculated conduction and valence bands of COF TpPa-1 (**hcb**) (b). The color in the computed band diagram encodes the fractional contribution of core orbital on the corresponding band.

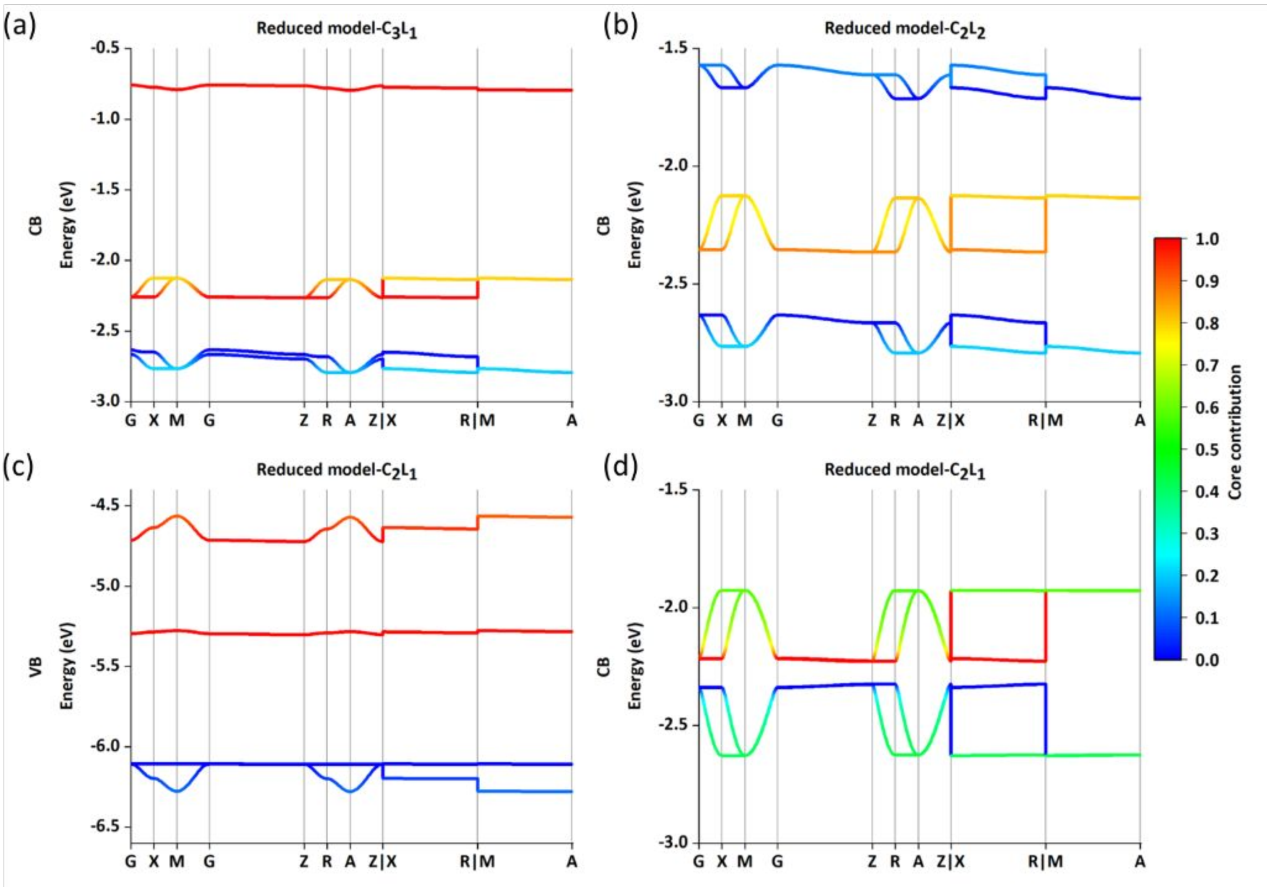

Figure S3. The conduction and valence bands of COF-366-Zn. The CB of the increase orbitals of core (a) and linker (b) from the partitioning in **Fig. 3**; the CB (c) and VB (d) with the parameters of C_2L_1 from another partitioning in **Fig. S10** (blue one). The color map of band denotes the contribution from the core and linker.

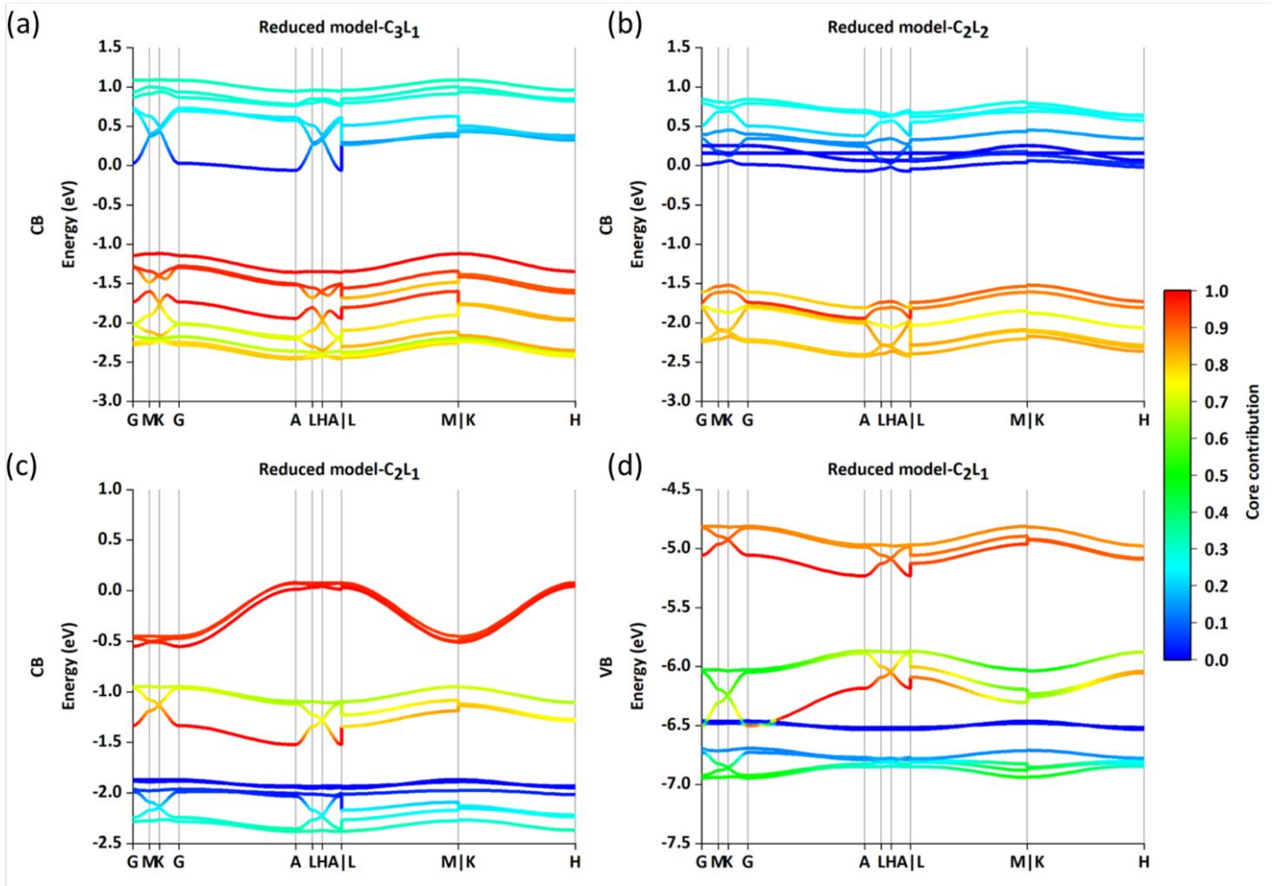

Figure S4. The conduction and valence bands of dual-pore COF. The CB of the increase orbitals of core (a) and linker (b) from the partitioning in **Fig. 4**; the CB (c) and VB (d) with the parameters of C2L1 from another partitioning in **Fig. S11** (blue one). The color map of band denotes the contribution from the core and linker.

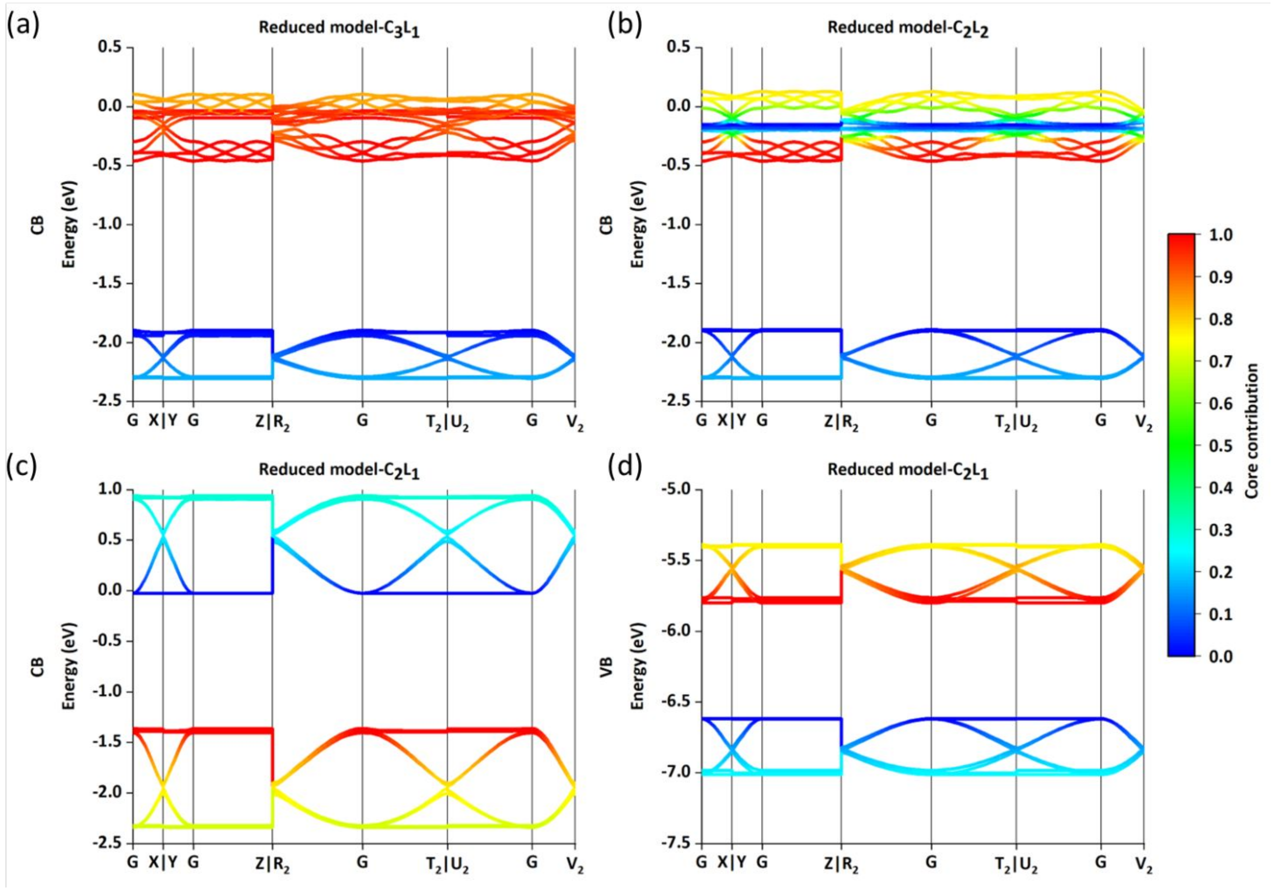

Figure S5. The conduction and valence bands of COF-300. (a) and (b) represent the CB of the increase orbitals of core (C_3L_1) and linker (C_2L_2) from the partition in **Fig. 5**; (c) and (d) are the CB and VB with the parameters of C_2L_1 from another partitioning in **Fig. S12** (blue one).

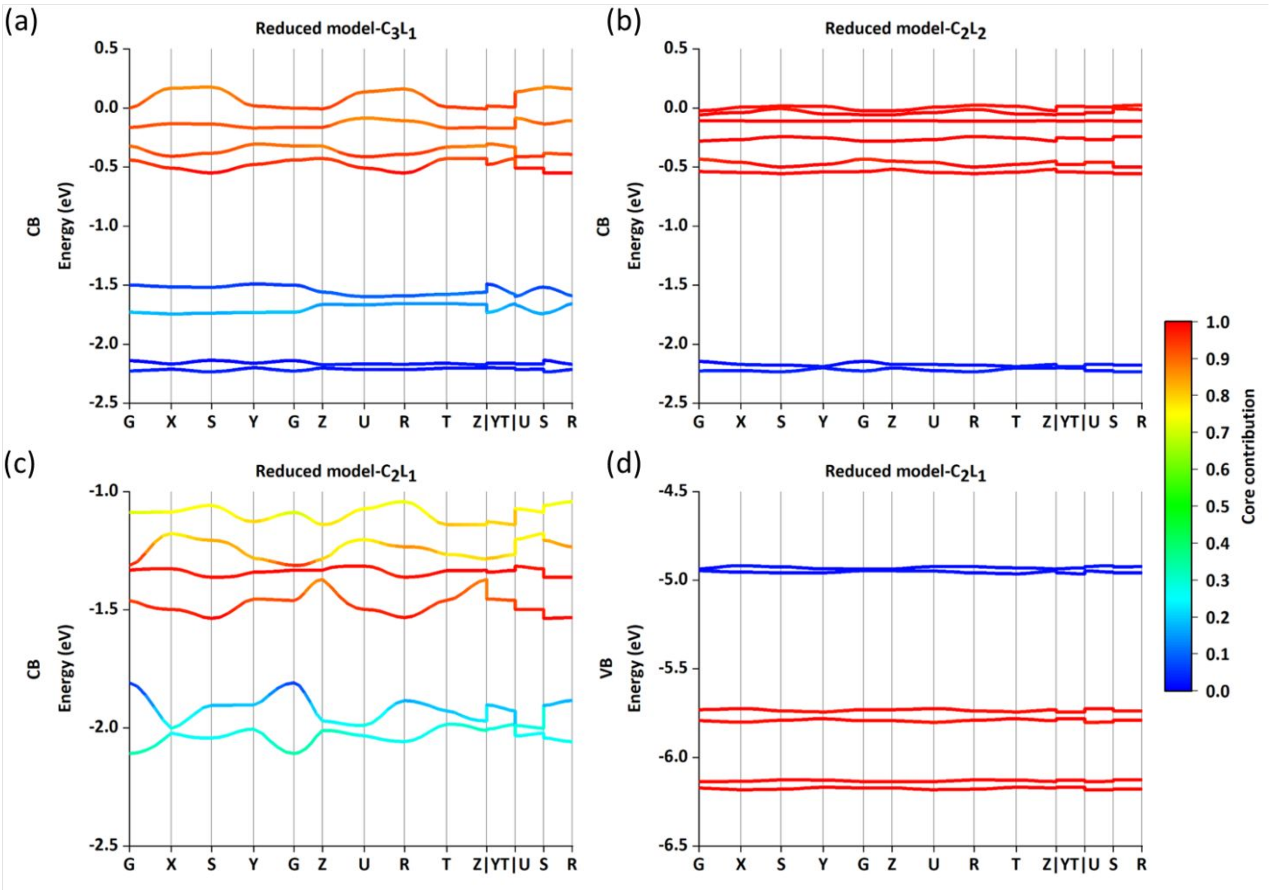

Figure S6. The conduction and valence bands of 3D-py-COF. he CB of the increase orbitals of core (a) and linker (b) from the partitioning in **Fig. 6**; the CB (c) and VB (d) with the parameters of C_2L_1 from another partitioning in **Fig. S13** (blue one). The color map of band denotes the contribution from the core and linker.

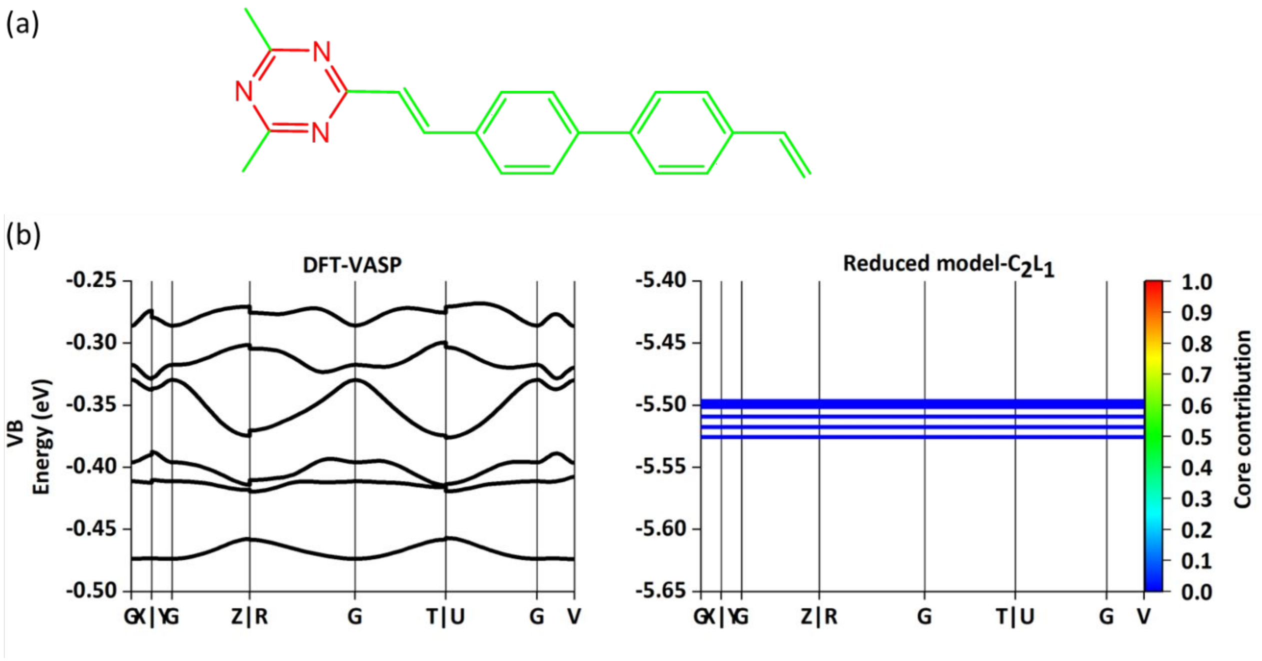

Figure S7. The valence bands (b) of COF-701 from VASP and reduced model with parameter C_2L_1 from the partitioning core and linker (a), respectively.

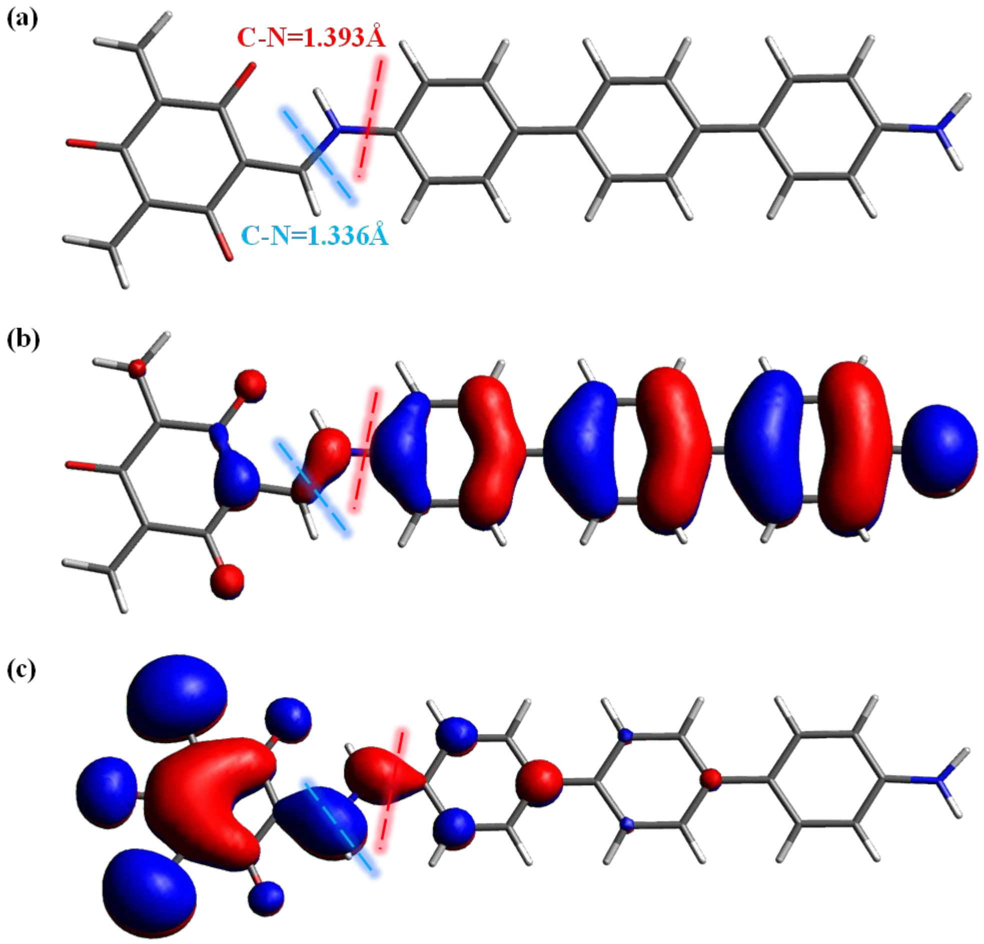

Figure S8. The different partitioning type of TP-COF. (a) is the geometrical structure of dimer of connected fragments, (b) is the HOMO and (c) is the LUMO. The red dotted line represents the best partition for the well fitted CB and VB in **Fig. 2** while blue one is the alternative partition.

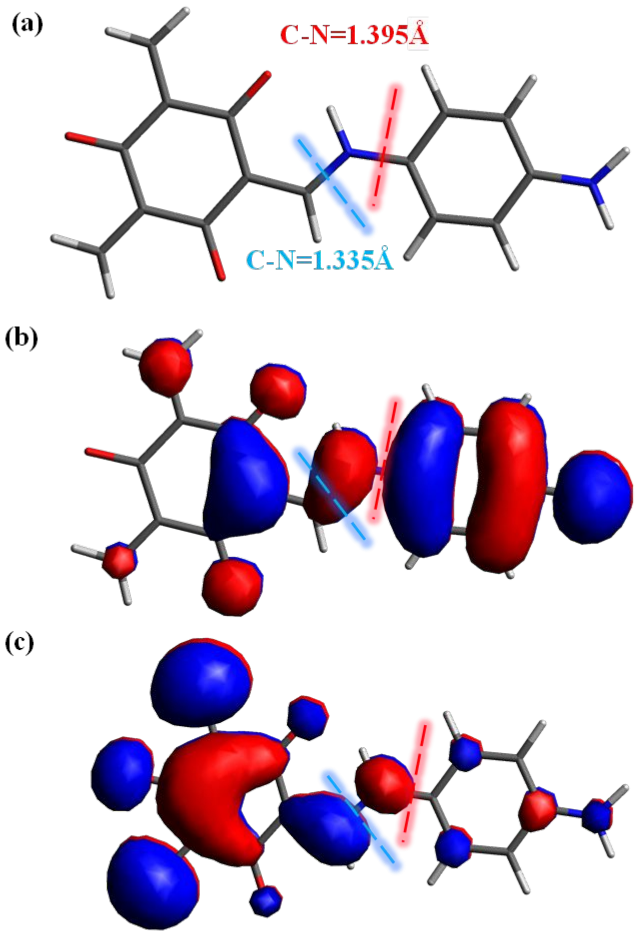

Figure S9. The different partitioning type of TpPa-1. (a) is the geometrical structure of dimer of connected fragments, (b) is the HOMO and (c) is the LUMO. The red dotted line represents the best partition for the well fitted CB and VB in **Fig. S2** while blue one is the alternative partition.

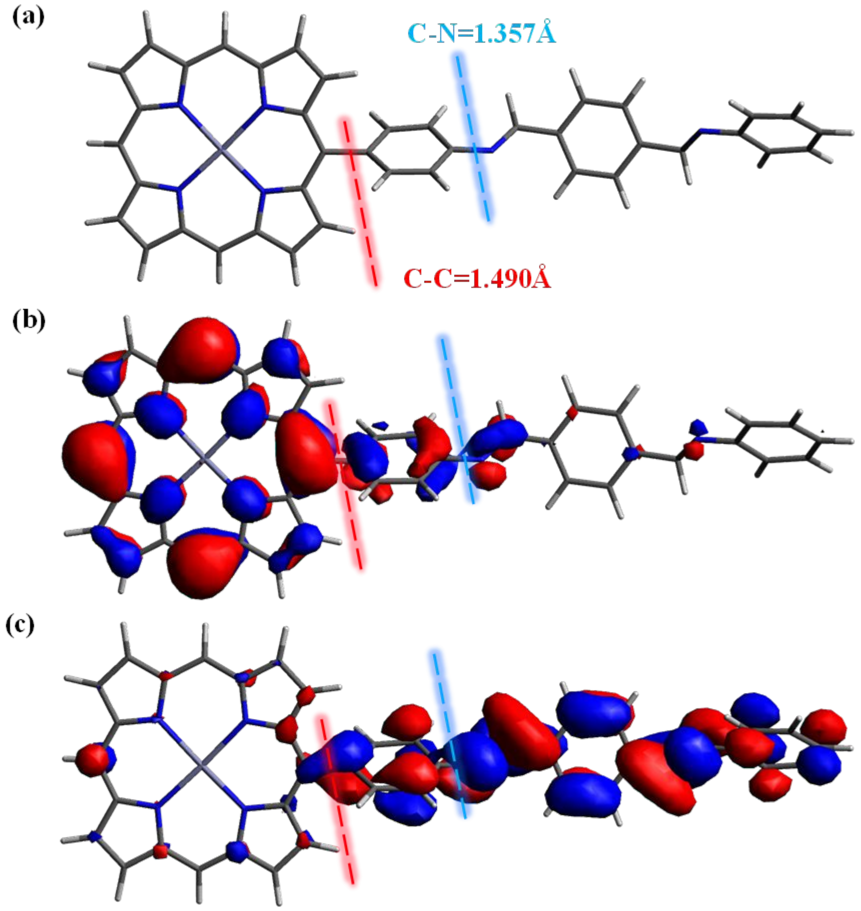

Figure S10. The different partitioning type of COF-366-Zn. (a) is the geometrical structure of dimer of connected fragments, (b) is the HOMO and (c) is the LUMO. The red dotted line represents the best partition for the well fitted CB and VB in **Fig. 3** while blue one is the alternative partition.

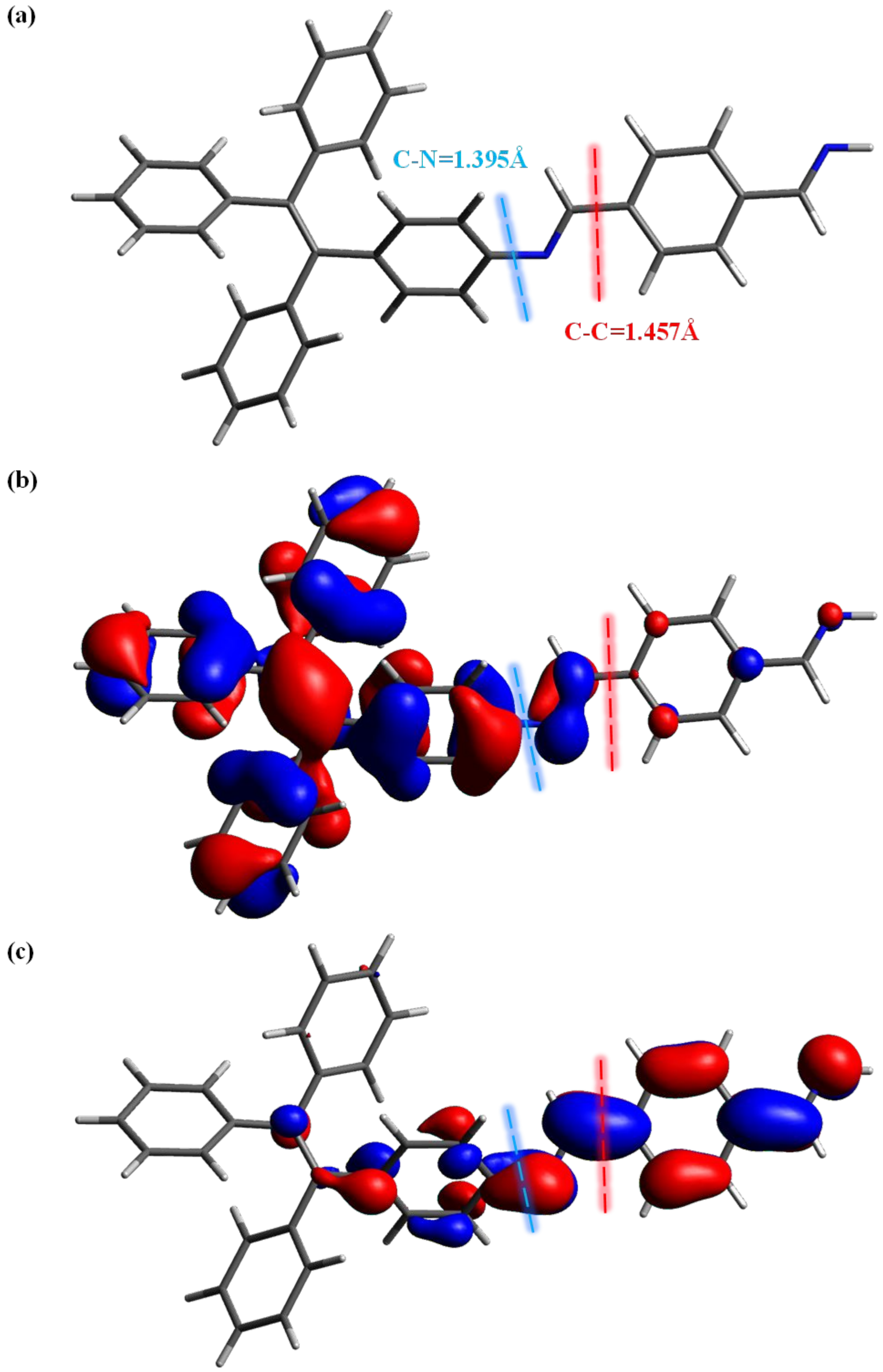

Figure S11. The different partitioning type of dual-pore COF. (a) is the geometrical structure of dimer of connected fragments, (b) is the HOMO and (c) is the LUMO. The red dotted line represents the best partition for the well fitted CB and VB in **Fig. 4** while blue one is the alternative partition.

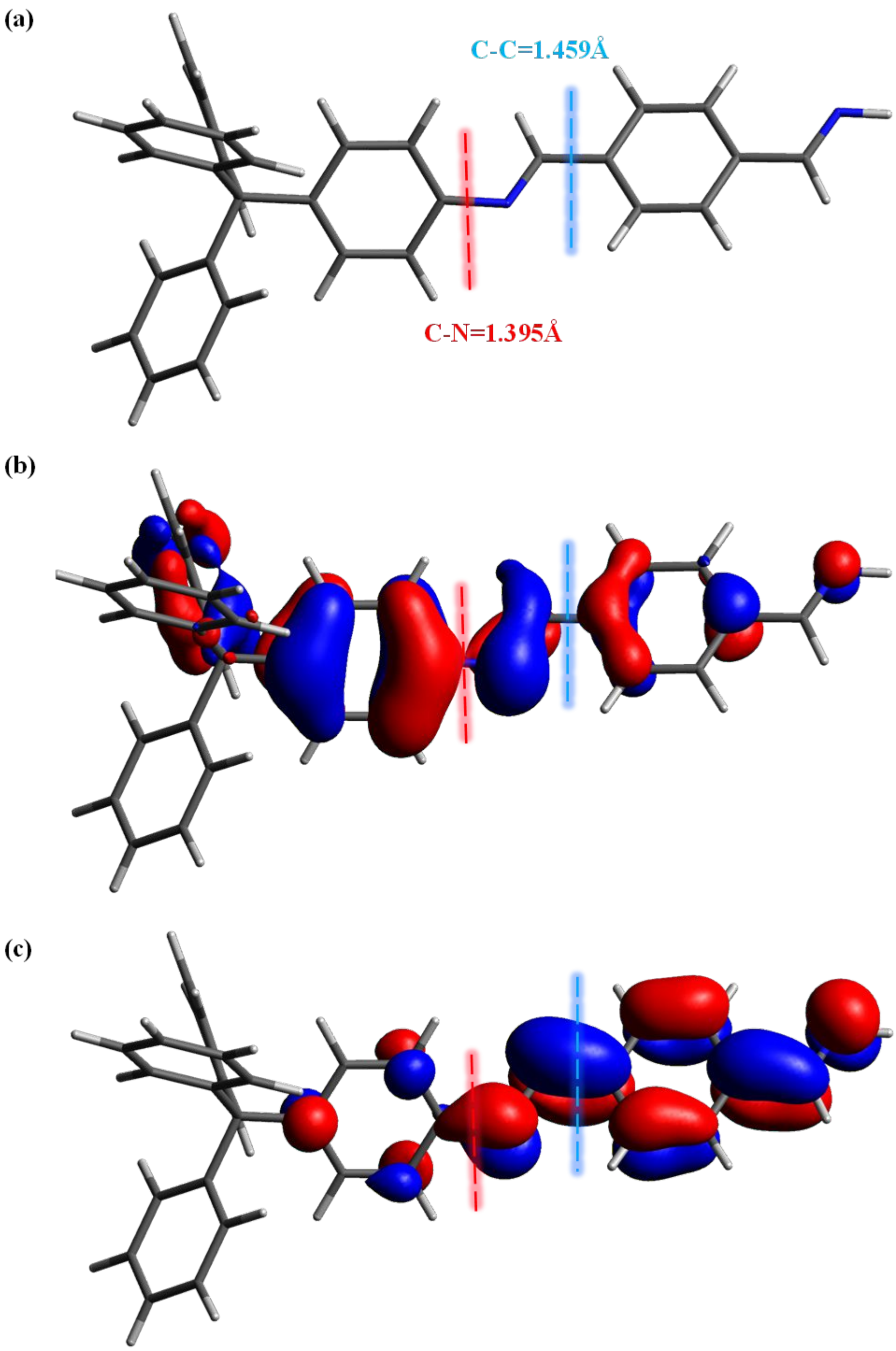

Figure S12. The different partitioning type of COF-300. (a) is the geometrical structure of dimer of connected fragments, (b) is the HOMO and (c) is the LUMO. The red dotted line represents the best partition for the well fitted CB and VB in **Fig. 5** while blue one is the alternative partition.

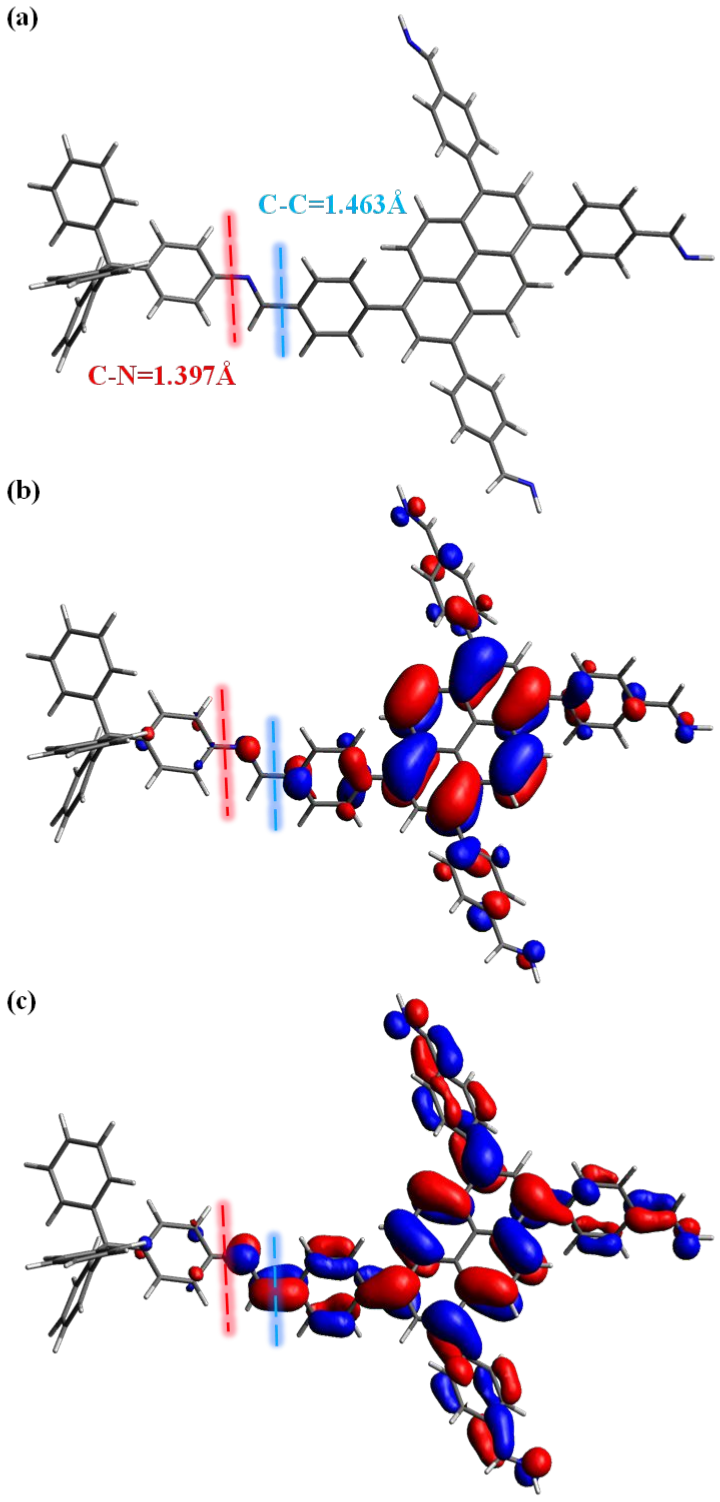

Figure S13. The different partitioning type of 3D-py-COF. (a) is the geometrical structure of dimer of connected fragments, (b) is the HOMO and (c) is the LUMO. The red dotted line represents the best partition for the well fitted CB and VB in **Fig. 6** while blue one is the alternative partition.

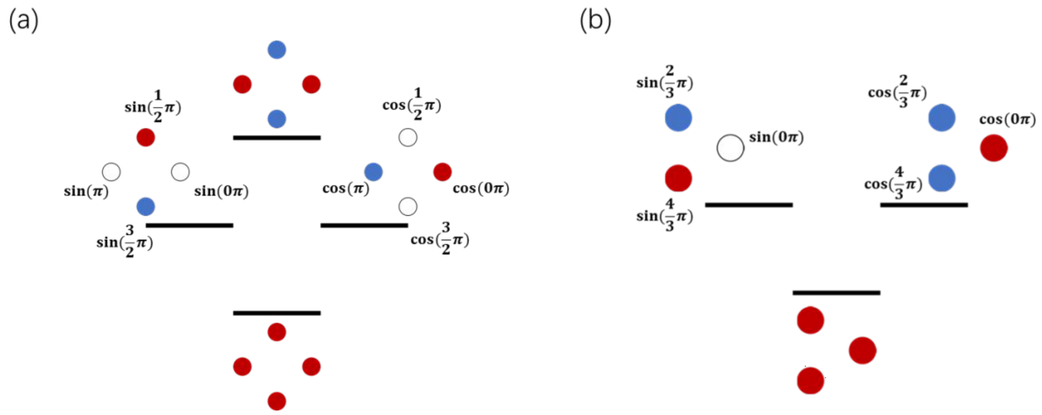

Figure S14. Orbitals illustration of (a) 4-member ring and (b) 3-member ring systems. Here $\cos\theta/\sin\theta$ are orbital coefficients (not normalised) based on the Hückel theory.

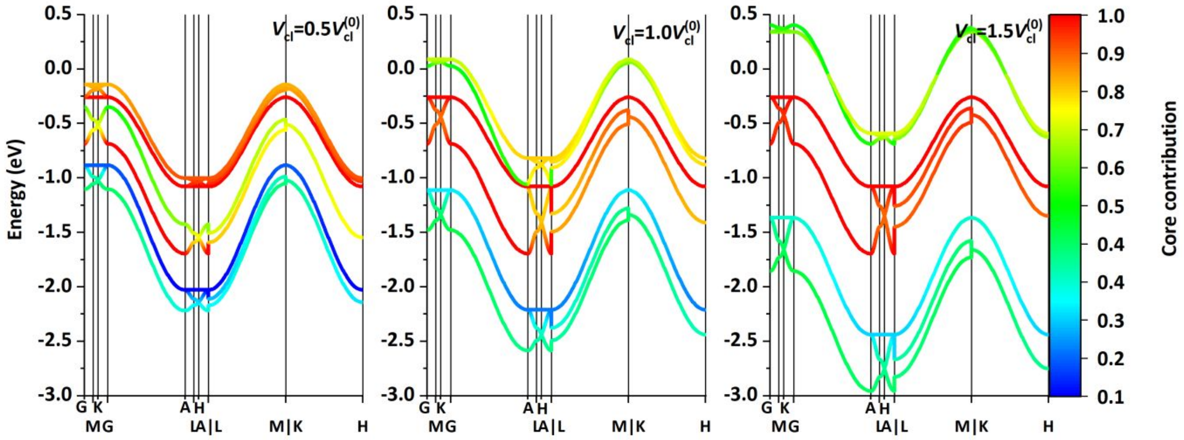

Figure S15. Projected band structure results for conduction bands of TP-COF using the simplest model with the change of core-linker couplings (blue in **Fig. 2(b)**). Colour presents the contribution of core orbitals in bands.

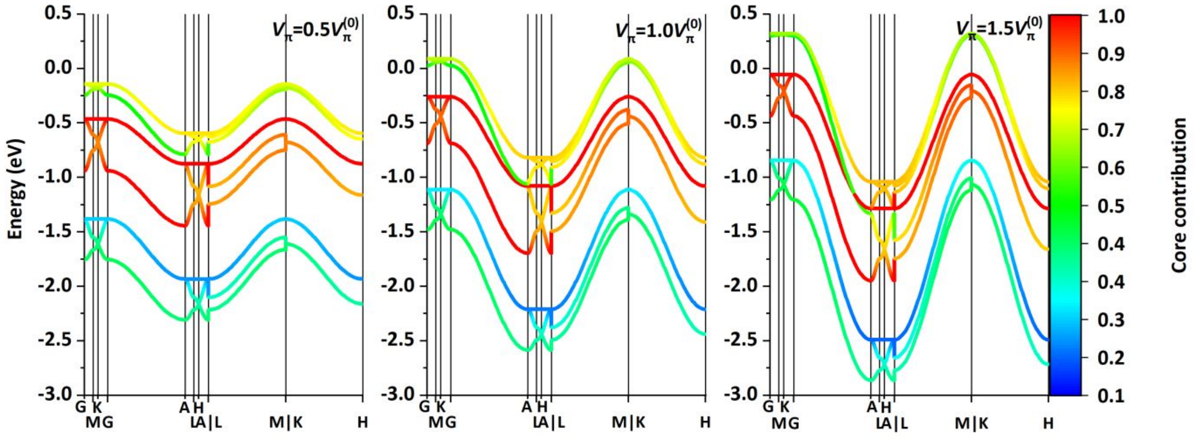

Figure S16. Projected band structure results for conduction bands of TP-COF using the simplest model with the change of $\pi-\pi$ couplings (red in **Fig. 2(b)**). Colour presents the contribution of core orbitals in bands.

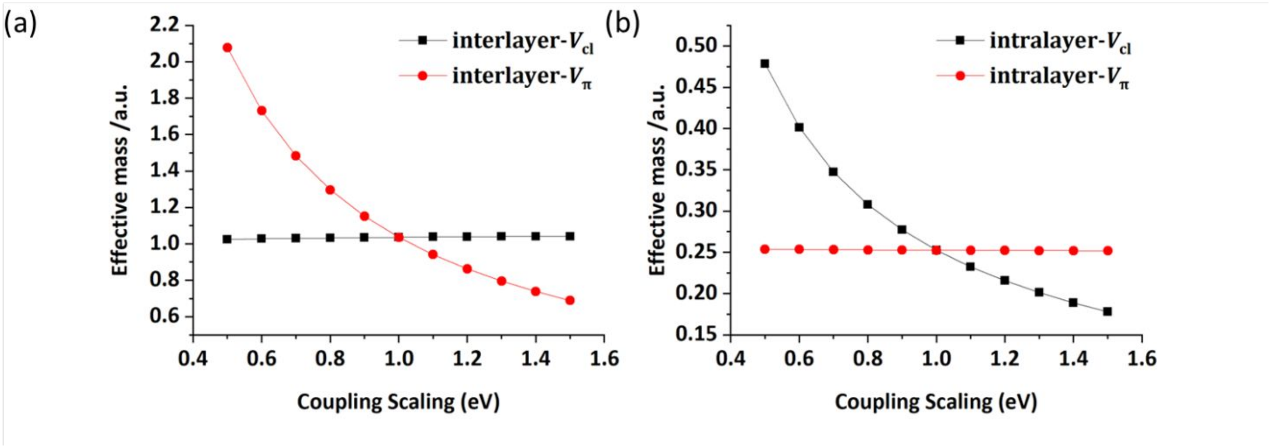

Figure S17. Effective mass for (a) interlayer (A[0.0, 0.0, 1/2] -> Γ[0.0, 0.0, 0.0] k path) and (b) intralayer (A[0.0, 0.0, 1/2] -> H[1/3, 1/3, 1/2] k path) electronic transport with coupling change. Here, V_{cl} for core-linker coupling (blue in **Fig. 2(b)**) and V_π for $\pi-\pi$ couplings (red in **Fig. 2(b)**).
